# p73 regulates ependymal planar cell polarity by modulating actin and microtubule cytoskeleton

**DOI:** 10.1038/s41419-018-1205-6

**Published:** 2018-12-05

**Authors:** Sandra Fuertes-Alvarez, Laura Maeso-Alonso, Javier Villoch-Fernandez, Merit Wildung, Marta Martin-Lopez, Clayton Marshall, Alberto J. Villena-Cortes, Inmaculada Diez-Prieto, Jennifer A. Pietenpol, Fadel Tissir, Muriel Lizé, Margarita M. Marques, Maria C. Marin

**Affiliations:** 1 Instituto de Biomedicina (IBIOMED) and Departamento de Biología Molecular, Universidad de León, Campus de Vegazana, 24071 León, Spain; 2 Molecular and Experimental Pneumology Group, Clinic for Cardiology and Pneumology, University Medical Center, 37077 Göttingen, Germany; 3 Institute of Molecular Oncology, Clinic for Cardiology and Pneumology, Department of Pneumology, University Medical Center Göttingen, Göttingen, Germany; 4 Department of Biochemistry and Vanderbilt-Ingram Cancer Center, Vanderbilt University and Vanderbilt University Medical Center, Nashville, TN 37232 USA; 5 Departamento de Medicina, Cirugía y Anatomía Veterinaria, Universidad de León, Campus de Vegazana, 24071 León, Spain; 6 Developmental Neurobiology, Institute of Neuroscience, Universite Catholique de Louvain, Avenue E. Mounier, 73, Box B1.73.16, B1200 Brussels, Belgium; 7 Instituto de Desarrollo Ganadero (INDEGSAL) and Departamento de Producción Animal, Universidad de León, Campus de Vegazana, 24071 León, Spain

## Abstract

Planar cell polarity (PCP) and intercellular junctional complexes establish tissue structure and coordinated behaviors across epithelial sheets. In multiciliated ependymal cells, rotational and translational PCP coordinate cilia beating and direct cerebrospinal fluid circulation. Thus, PCP disruption results in ciliopathies and hydrocephalus. PCP establishment depends on the polarization of cytoskeleton and requires the asymmetric localization of core and global regulatory modules, including membrane proteins like Vangl1/2 or Frizzled. We analyzed the subcellular localization of select proteins that make up these modules in ependymal cells and the effect of *Trp73* loss on their localization. We identify a novel function of the *Trp73* tumor suppressor gene, the TAp73 isoform in particular, as an essential regulator of PCP through the modulation of actin and microtubule cytoskeleton dynamics, demonstrating that *Trp73* is a key player in the organization of ependymal ciliated epithelia. Mechanistically, we show that p73 regulates translational PCP and actin dynamics through TAp73-dependent modulation of non-musclemyosin-II activity. In addition, TAp73 is required for the asymmetric localization of PCP-core and global signaling modules and regulates polarized microtubule dynamics, which in turn set up the rotational PCP. Therefore, TAp73 modulates, directly and/or indirectly, transcriptional programs regulating actin and microtubules dynamics and Golgi organization signaling pathways. These results shed light into the mechanism of ependymal cell planar polarization and reveal p73 as an epithelial architect during development regulating the cellular cytoskeleton.

## Introduction

The specific orientation of cells within the plane of the tissue, named planar cell polarity (PCP), is an essential feature of animal tissues^1^. PCP signaling is required for polarized beating of motile cilia in a variety of tissues^2^, including multiciliated ependymal cells (ECs), which carpet the wall of the lateral ventricles^3,4^. ECs, which are established perinatally from radial glial cells (RGCs) in a multistep process, display two types of PCP, rotational (rPCP) and translational (tPCP). display two types of PCP, rotational (rPCP) and translational (tPCP), which are established perinatally from radial glial cells (RGCs) in a multistep process^5^. rPCP is defined by the unidirectional orientation of the motile cilia within the cell and is coordinated at tissue-level. tPCP initiates in RGCs when their primary cilium is asymmetrically displaced^3,6^. Postnatally, immature multiciliated ECs displace their cilia clusters toward the anterior apical surface^4^.

PCP is regulated by asymmetric signaling through core and global regulatory modules. PCP-core module includes Frizzled (Fzd3, 6), Van Gogh-like (Vangl1/2), cadherin epidermal growth factor (EGF)-like laminin G-like seven-pass G-type receptor (Celsr1-3), Dishevelled (Dvl1-3), and Prickle (Pk1-4). In rodents, asymmetric localization of the PCP-core complexes is required for the rotational orientation of basal bodies (BBs) in multiciliated cells^3,7–12^. There is an initial polarization of the apical microtubule (MT) cytoskeleton, which induces the asymmetric distribution of PCP-core complexes at apical junctions^13^. These, in turn, will communicate polarity information to ciliary BBs distributed within the sub-apical cytoskeleton networks^12,14^. PCP-core signaling regulates the rotational, but not the translational, polarity in ECs^15^. In these cells, activation of the actin-binding protein non-muscle myosin-II (NMII) by the myosin light chain kinase (MLCK) is essential for tPCP establishment^15^. Thus, both actin and MT networks are key factors for setting up PCP in ECs.

The causal relationship between ciliogenesis and PCP signaling is not fully deciphered^16^. In this regard, the *Trp73* gene is a key player in the organization of multiciliated cells^17–19^ and in EC polarity^17,20^. p73 belongs to the p53 family of transcription factors and generates functionally different TA and DNp73 isoforms^21^. Neither p73 mechanisms underlying PCP regulation nor the responsible p73-isoform, have been addressed.

In this work we analyzed the subcellular localization of PCP-regulatory proteins in ECs and the effect of *Trp73* gene loss on their localization. p73 deficiency resulted in loss of PCP-core signaling complex asymmetry and lack of translational and rotational polarity in ECs. We demonstrate that p73 regulates PCP, at least in part, through TAp73-modulation of NMII activity via *MLCK* transcriptional regulation and regulation of MT dynamics signaling pathways.

## Materials and methods

### Animal care, genotyping, and isolation of wholemounts

All animal experiments were carried out in accordance with European (European Council Directive 2010/63/UE) and Spanish regulations (RD 53/2013) as well as institutional animal ethical guidelines. Mice heterozygous for *Trp73* on a mixed background C57BL/6 × 129/svJae^22^ were backcrossed to C57BL/6, at least five times, to enrich for C57BL/6 background. Heterozygous animals were crossed to obtain the *Trp73*−/− mice (p73KO from now on). DNp73 and TAp73 mutant mice have been described before^23,31^. Male and female mice, obtained from multiple litters, were used for the experiments. Genotyping of animals was performed by PCR analysis as described before^22–24^.

For wholemount (WM) isolation, animals were euthanized and brains were dissected and placed in cold 0.1 M phosphate-buffered saline. Dissections of the lateral wall of the lateral ventricles were performed under the stereomicroscope as described before^6^.

### Cell culture

Mouse wild type (WT) and p73KO-induced pluripotent stem cells (iPSCs) were cultured on feeder cells as described before^25^. For transfection assays, iPSCs were subcultured once without feeders in plates coated with Matrigel (Corning, NY, USA) and then seeded (3.7 × 10^4^ cells/cm^2^) on coverslips (coated with Matrigel). Cells were transfected with the indicated plasmids using Lipofectamine™2000 Transfection Reagent (Invitrogen, Carlsbad, CA, USA) and after 24 h were fixed with 3.7% paraformaldehyde (PFA) for 15 min at room temperature (RT). For MLCK recovery assays, p73KO-iPSCs were transfected with a constitutively active MLCK expression vector (pSLIK-CA-MLCK) (Addgene plasmid # 84647). After 24 h cells were treated with doxycycline (400 ng/mL) to induce MLCK expression and were analyzed 24 h later.

HA-TAp73β-Saos-2-Tet-On cells^26^, (inducible TAp73-Saos-2 cells from now on) kindly provided by Dr. Karen Vousden (Cancer Research UK Beatson Institute, Glasgow), were cultured in Dulbecco’s modified Eagle’s medium supplemented with 10% fetal bovine serum (FBS), 2 mM l-glutamine, and 100 U/mL penicillin–0.1 mg/mL streptomycin. To induce TAp73 expression, cells seeded on coverslips for 24 h (3 × 104 cells/cm^2^) were treated with 2.5 μg/mL of doxycycline for 48 h^27^. Then, fixation was carried out with 3.7% PFA at RT during 15 min.

The human cell line HCT116 was cultured in RPMI supplemented with 10% FBS, 2 mM l-glutamine, and 100 U/mL penicillin–0.1 mg/mL streptomycin.

### Luciferase assays

HCT116 cells were seeded in 24-well plates (3 × 105 cell/cm^2^) and transfected with 0.125 μg of the luciferase reporter pGL3-hMLCK(1Kb)-*luc* (kindly provided by Dr. Jerrold Turner, University of Chicago)^28^, 0.0625 μg pRLNull renilla, and 0.6 or 0.8 μg of the indicated expression vectors. Transfection was performed using X-tremeGENE^TM^ HP DNA Transfection Reagent (Roche, Basel, Switzerland).

Saos-2 were transfected with empty pcDNA3.1 or containing p73 isoforms and a Firefly luciferase reporter containing the putative WT TAp73-binding sequence (WT, Table S2) as identified by chromatin immunoprecipitation (ChIP)-seq^29^ or the same sequence lacking the predicted TAp73-binding motives (Mutated, Table S2). Transfection efficiency was controlled by co-transfecting a Renilla TK luciferase vector. Luciferase activity was always assayed 24 h after transfection using Dual luciferase assay. Firefly luciferase values were normalized to the corresponding Renilla luciferase levels.

### Western blot analysis

Western blotting was performed as previously described^30^ and is detailed in the Supplementary Experimental Procedures.

### Immunostaining and image analysis

Immunostaining protocols are described in the Supplementary Experimental Procedures. WM samples were analyzed using the Zeiss LSM800 and Olympus FluoView FV10i confocal laser scanning microscopes. iPSCs and inducible-TAp73-Saos-2 samples were analyzed using a Zeiss LSM800 Confocal Laser Scanning Microscope and a Nikon Eclipse TE2000 Confocal Microscope. Confocal *z*-stack images were taken in all cases. Images were processed using ZEN blue software, FV10-ASW 2.1 viewer software, EZ-C1 software, and ImageJ software.

### Morphometric analysis

For the quantification of BB displacement in RGCs (P1) and in immature ECs (P15 or P21), at least three independent mice from each genotype were considered, counting at least three non-overlapping fields (50 μm × 50 μm) of each animal. Image analysis was performed using ImageJ software, quantifying the displacement by measuring a vector from the cell center to the BB (P1) or to the patch centroid (P15/P21).

For the quantification of number and intensity of p-MLC2 membrane dots, five independent mice from each genotype were considered, counting six non-overlapping fields (1 cell/field; ±20 μm × 20 μm) of each animal. Single plane images were obtained from *z*-stacks. Image analysis was performed using ZEN blue software, analyzing number and intensity of p-MLC dots in membrane region.

For the quantification of BB docking, three independent adult mice from each genotype were considered, counting two non-overlapping fields of each animal (50 μm × 50 μm; ±40 cells per animal). Orthogonal view of *z*-stack image analysis was performed with ZEN blue software, counting the number of cells with BBs aligned with the plasma membrane and cells with BBs scattered within the cytoplasm.

For the quantification of rotational PCP, a combined staining of FGFR1 Oncogene Partner (FOP) and γ-tubulin was used to delineate cilia polarity^3^. The FOP signal is adjacent to that of γ-tubulin at the side opposite to the basal foot. A vector that represents the direction of the individual cilia beat was defined by the direction from FOP (blue) to γ-tubulin (red) dots, using ImageJ software and all data were transformed to positive angles. The angle of polarity formed by this vector with respect to a horizontal axis was drawn. For each cell, a mean rotational angle and the circular standard deviation (CSD) were calculated with Oriana software. Five independent mice from each genotype were considered, counting three non-overlapping fields of each animal (50 μm × 50 μm; ±21 cells per animal).

For the quantification of Vangl2 and Frizzled localization at the plasma membrane, confocal *z*-stack images were taken before and after transiently transfecting the indicated expression vectors into p73KO-iPSCs. Single-plane images were obtained from *z*-stacks. Gaussian Blur filter (*σ* = 10) was applied and then images were binarized using automatic threshold. The midline along the membrane was created using Skeletonize Option. This network was dilated (15 iterations) to create the plasma membrane selection. which was overlapped with Vangl2 and Frizzled3 staining and the mean fluorescence intensity was measured.

### RNA isolation and real-time quantitative reverse transcription-PCR analysis

Total RNA from cultured cells or dissected WMs was isolated using TRI reagent (Ambion, TX, USA). Kidney, bladder, and retina total RNA were isolated using RNeasy mini Kit (Qiagen, Hilden, Germany) following the manufacturer’s instructions. cDNA was synthesized using up to 2 μg of total RNA and the High Capacity RNA-to-cDNA kit (Applied Biosystems, Carlsbad, CA, USA). Gene expression was detected by real-time quantitative reverse transcription-PCR (qRT-PCR) in a StepOnePlus Real-Time PCR System (Applied Biosystems, Carlsbad, CA, USA) using FastStart Universal SYBR Green Master (Roche, Basel, Switzerland). qRT-PCR conditions were described before^31^. mRNA expression levels were expressed as 2^ΔCt^ (ΔCt = Ct internal reference − Ct gene), normalized to 18S mRNA expression. Primer sequences were as follows: *18S* mouse gene^32^: F 5′-AGTTCCAGCACATTTTGCGAG-3′ and R 5′-TCATCCTCCGTGAGTTCTCCA-3′; *Mlck* mouse gene (Primer Bank Database ID: 29650205a1): F 5′-TGGGGGACGTGAAACTGTTTG-3′ and R 5′-GGGGCAGAATGAAAGCTGG-3′; *TAp73* mouse gene F 5′-GCACCTACTTTGACCTCCCC-3′ and R 5′-GCACTGCTGAGCAAATTGAAC-3′, and *Eb3* mouse gene F 5′-ACTAGAACACGAGTACATCCACA-3′ and R 5′-CCATCATAGTTTGCGTCAAAGA-3′. *18S* human gene: F 5′-GGCGCCCCCTCGATGCTCTTA-3′ and R 5′-GCTCGGGCCTGCTTTGAACAC-3′; *MLCK* human gene: F 5′-CACCGTCCATGAAAAGAAGAGTAG-3′ and R 5′-GAGAGGCCCTGCAGGAAGATGG-3′; *TAp73* human gene: F 5′-GCACCTACTTCGACCTTCCC-3′ and R 5′-GTGCTGCTCAGCAGATTGAAC-3′; *EB3* human gene: F 5′-GCCAATGATGCCTGAGATAACAG-3′ and R 5′-GCAGAGGGTTGTAATCCTTTCCA-3′.

### Sequence analysis and ChIP

In silico prediction of putative p73-response elements within the mouse *Mlck* gene was performed using the open-access database for eukaryotic transcription factor-binding profiles JASPAR^33^. Matrix models MA0861.1 and MA0525.1 were applied for the mouse *Mlck* gene and for the analysis of the ChIP-seq peak detected in the human *MLCK* gene, respectively. Only the sites with a score over 9.0 were selected for further in vitro analysis.

ChIP analyses were carried out as previously described^25^ and are detailed in the Supplementary Experimental Procedures.

### Gene ontology analysis

Gene ontology (GO) analysis was carried out using selected genes from GSE15780. DAVID Bioinformatics Resources 6.8^34,35^ and PANTHER™ GO slim (version 13.1, released 2018-02-03)^36^ were used for the analysis. The results were plotted using the ggplot2 library from R (3.5.1) Core Team (2014), a language and environment for statistical computing. R Foundation for Statistical Computing, Vienna, Austria. URL http://www.R-project.org/.

### Statistical analysis

**Fig. 1:**
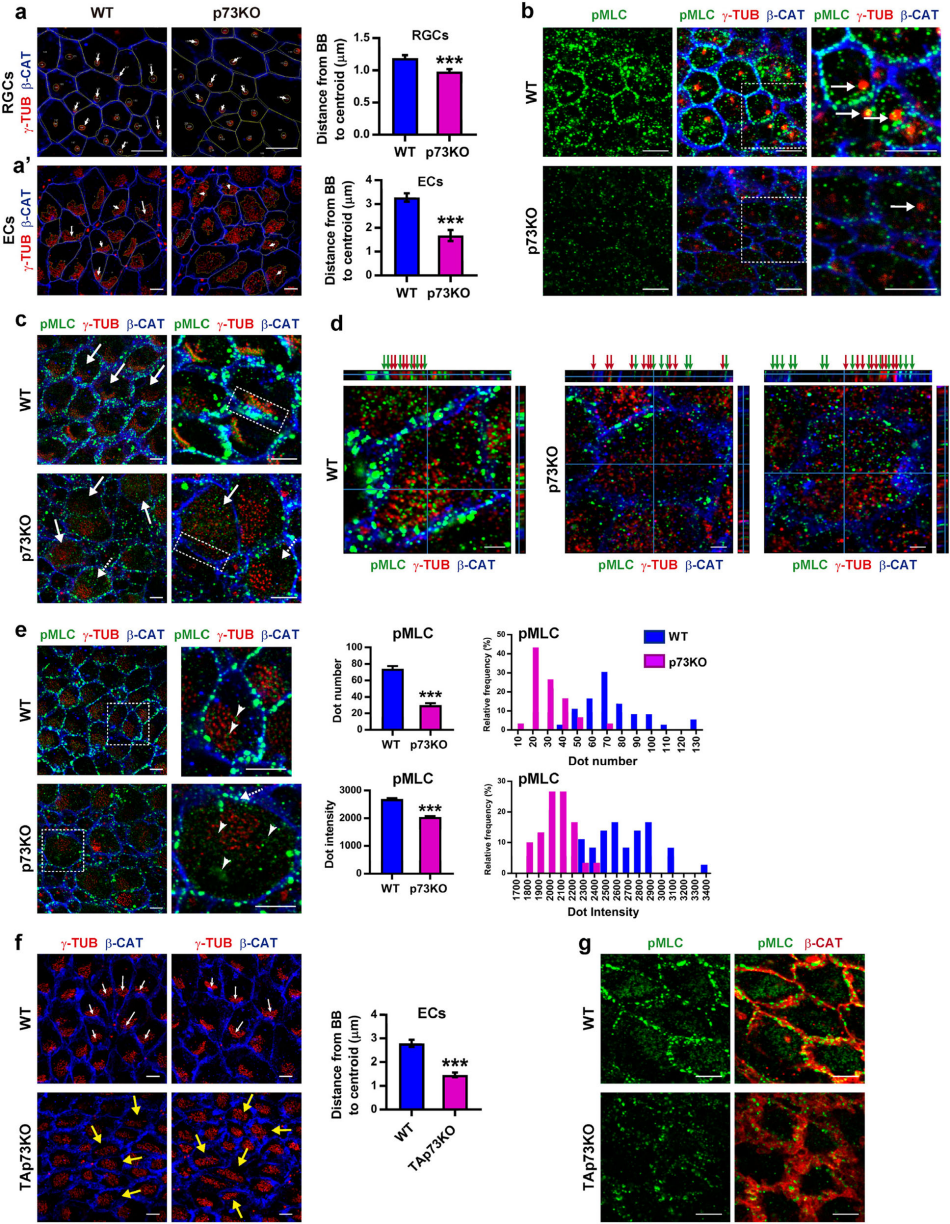
p73 coordinates tPCP establishment in RGC and ECs through the regulation of polarized NMII activity. **a**–**g** Wholemounts (WMs) from WT, p73KO (**a**–**e**) or TAp73KO (**f**, **g**) LW were stained with the indicated antibodies and confocal *z*-stack images were generated. WMs were stained with anti-γ-tubulin (red, basal bodies), p-MLC (green), and β-catenin (blue, cell contour, red in **g**). (**a**, **a′**) Comparative quantification of BB displacement in P1-RGCs (**a**) showing 10–30 mm^2^ apical surface or P15-ECs (**a′**) using ImageJ software. Scale bar: 5 µm. A vector of displacement was drawn from the cell center to the BB-cluster centroid. Bars represent mean values ± S.E. (RGCs: *n* = 24 cells from three mice per genotype; ECs: *n* = 15 cells from five mice per genotype; ***p < 0.001). **b**–**e** WMs from P1-RGCs (**b**) or P15-ECs (**c**–**e**) were stained with anti-p-MLC to detect activated NMII. p-MLC and primary cilia BB colocalization are indicated by arrows. Magnification insert at the right most panel. Scale bar: 5 µm. **c** BB-clusters crescents and orientation are indicated by arrows. p-MLC polarized membrane localization is marked by a dotted box. Scale bar: 5 µm. **d** Orthogonal projections show p-MLC signal (green arrows) adjacent to BB (red arrows). Scale bar: 2 µm. **e** Quantification of p-MLC dot number (upper panel) and average dot intensity (lower panel) at the plasma membrane. Scale bar: 5 µm. Right graphs represent the frequency distribution of those values in the cellular population. Bars represent mean values ± SE (*n* = 15 cells from five mice per genotype). **f**, **g** Analysis and quantification of BB-clusters displacement (**f**) and p-MLC localization (**g**) in P21 TAp73KO-ECs. **f** BB-cluster displacement in WT cells is depicted by white arrows and lack of coordination in BB-cluster displacement in p73KO cells by yellow arrows. Scale bar: 5 µm. **g** Scale bar: 5 µm

**Fig. 2:**
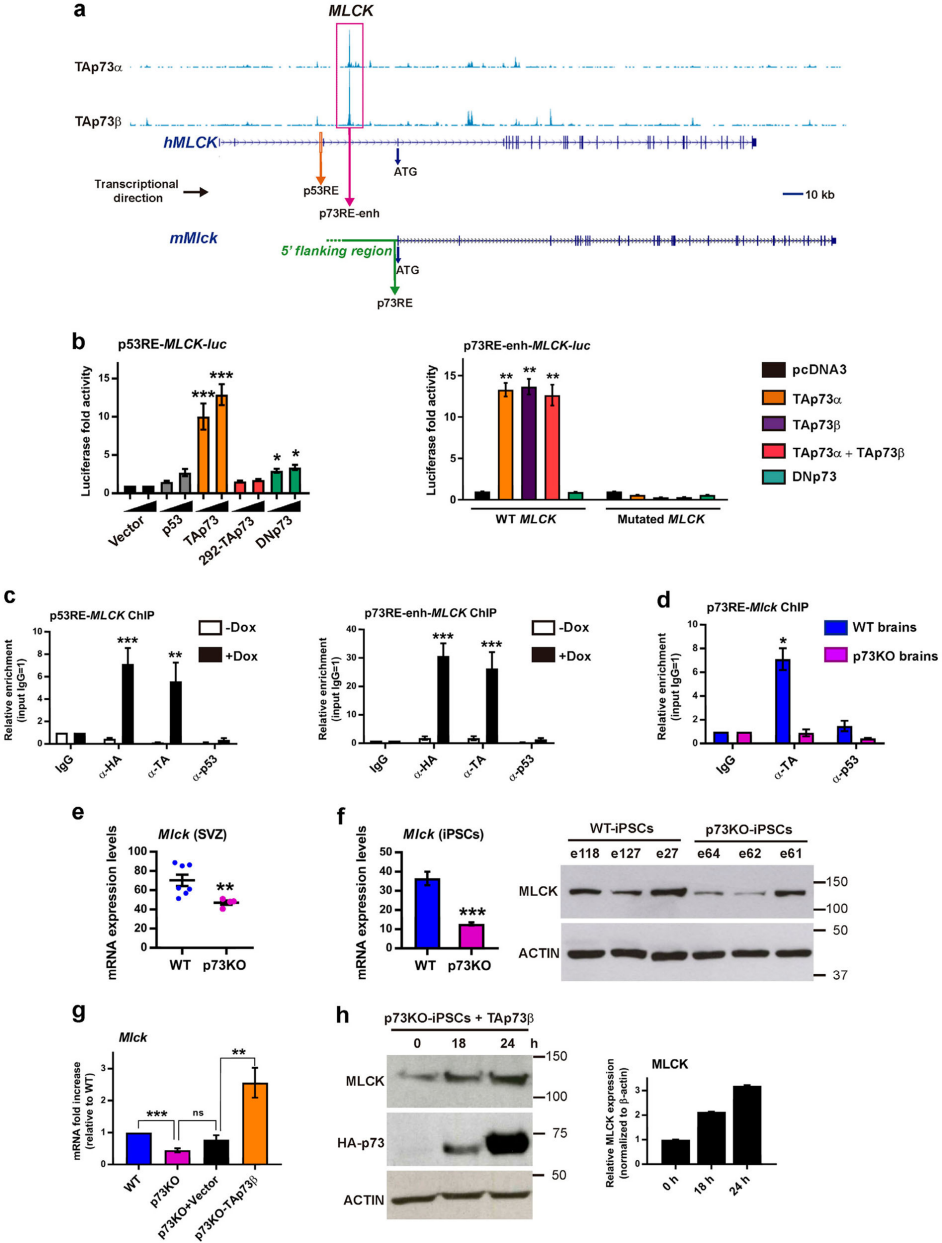
TAp73 transcriptionally regulates *Mlck* expression. **a** TAp73 ChIP-seq genome tracks of the human *MLCK* locus (NG_029111.1). The transcriptional direction is marked with an horizontal arrow. TAp73 peak matches to intronic sequence upstream the exon that contains the ATG (region 70,706–70,883 bp from NCBI Reference Sequence: NG_029111.1). Localization of the identified response elements (RE) is indicated by an orange arrow (p53RE) and a pink arrow (p73RE enhancer) in the human *MLCK* gene, and by a green arrow (p73RE) in the mouse *Mlck* gene. **b** Luciferase reporter assays were performed with the indicated expression vectors together with the reporter constructs pGL3-h*MLCK*(1Kb)-*luc*, containing the human *MLCK* regulatory region with the p53RE (left panel), or p73RE-enh-*MLCK*-*luc*, containing a minimal promoter and the putative p73 enhancer element (p73RE, right panel). Mutated control reporter contains the same DNA region but lacks the *TP73*-binding motifs. Three independent experiments per reporter were performed in in triplicate p53-deficient cell lines: HCT116 (p53RE-MLCK-*luc*) and Saos-2 (p73RE-enh-*MLCK*-*luc)*. Fold increase activity was calculated relative to control vector. Error bars represent standard error of the mean. Differences were considered significant when *p* < 0.05. **c**, **d** Direct binding of ectopic (**c**) or endogenous (**d**) TAp73 analyzed by ChIP assays that were carried out in inducible-TAp73-Saos2 cells (**c**, *n* = 3) or in mouse brain tissue (**d**, *n* = 3). The p53RE (**c**, left panel) and the p73RE enhancer element (**c**, right panel) of the h*MLCK* promoter region, or the p73RE of the proximal mouse *Mlck* promoter (**d**), were amplified by q-PCR. The data were normalized to input chromatin samples for each case and to IgG values = 1. Dox: doxycycline **e**, **f** Quantitative RT-PCR analysis of *Mlck* expression levels in WT and p73KO samples, from either ventricle LW of mice brains (**e**, *n* = 9 WT brains and *n* = 4 p73KO brains) or iPSC (**e**, *n* = 3 clones per genotype). **f** MLCK protein expression analysis in iPSCs by western blot. **g**, **h** Induction of *Mlck* analyzed by qRT-PCR (**g**) or western blot (**h**) in p73KO-iPSCs after ectopic expression of TAp73 or empty vector. Two independent transfection experiments were performed with two clones per genotype. qRT-PCR assays were repeated four times by duplicate. Bars represent mean values ± S.E. *p < 0.05, **p < 0.01, ***p < 0.001, n.s: non-significant

**Fig. 3:**
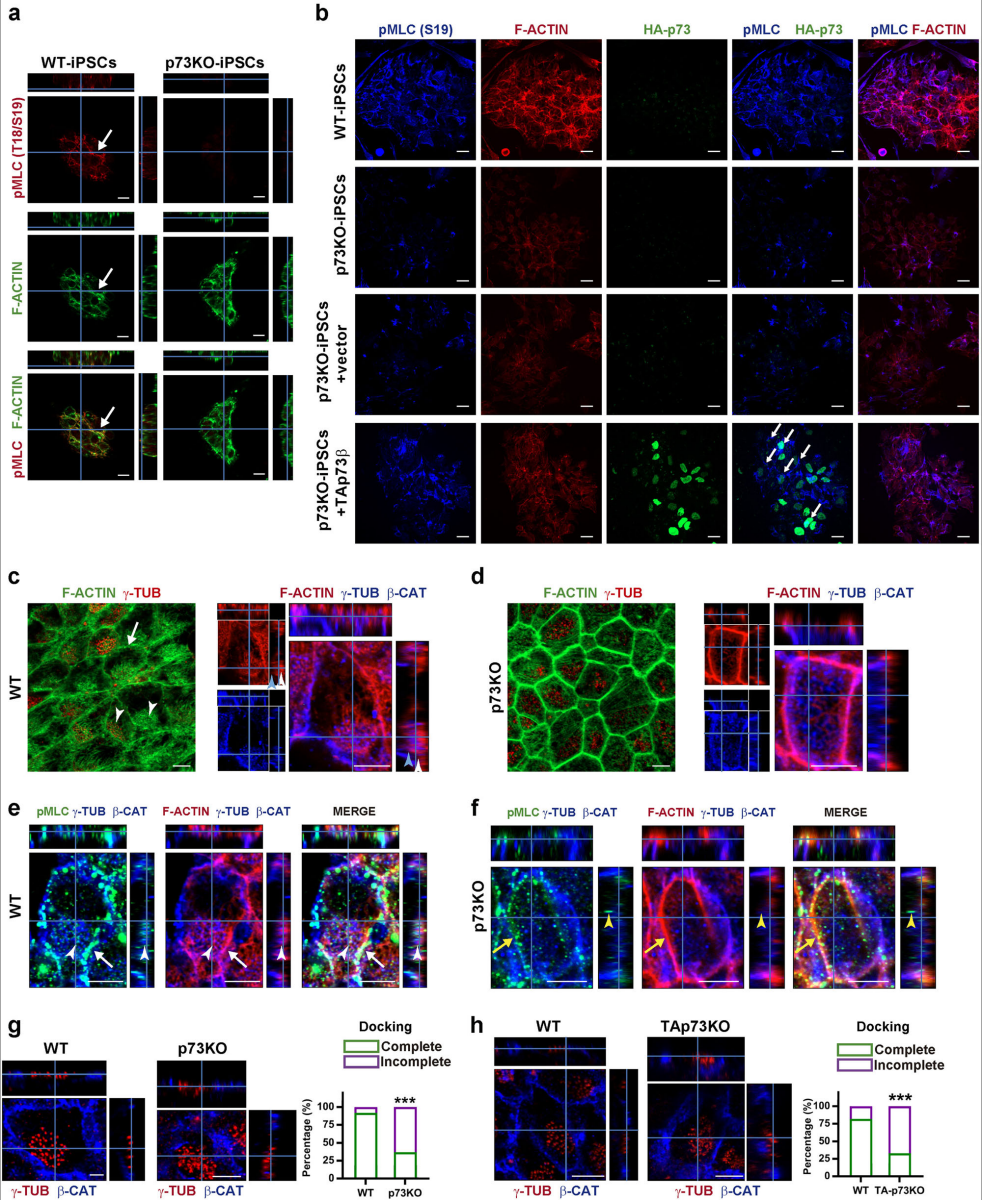
p73 regulation of the actin cytoskeleton and NMII activation is essential for the formation of sub-apical actin lattices and BB docking in ECs. **a**, **b** Detection of activated NMII and actin cytoskeleton in WT vs. p73KO-iPSC colonies (**a**), or after ectopic TAp73 expression in p73KO-iPSCs (**b**). **a** Orthogonal projections from WT and p73KO-iPSC where arrows indicate F-actin and p-MLC colocalization at intercellular junctions. Scale bar: 10 µm. **b** p-MLC colocalization with cortical actin bundles is recovered (arrows) upon TAp73 transfection, but not vector control. Scale bar: 10 µm. **c**–**f** Confocal images of WT (**c**, **e**) and p73KO (**d**, **f**) P15 WMs displaying as indicated: actin cytoskeleton (Phalloidin), p-MLC (green), and BBs alone (γ-tubulin, red) or with the cell membrane (β-catenin and γ-tubulin, blue). The white arrows point to cortical actin; the white arrowheads indicate apical actin lattices and blue arrowheads mark the sub-apical actin filaments. Scale bar: 5 µm. **e**, **f** p-MLC puncta associated with actin lattice around BBs or with plasma membrane are marked by white arrowheads and white arrows, respectively. **f** Yellow arrowheads and yellow arrows indicate the lack of such p-MLC association. Scale bar: 5 µm. (**g**, **g′**) BB docking was analyzed in WT and p73KO (**g**) or TAp73KO (**g′**) ECs. Lateral views of WM confocal images labeled with anti-γ-tubulin (red) and β-catenin (blue). Scale bar: 5 µm. Graphs represent the percentage of cells with correct docking (green bars) versus cells with incomplete alignment (purple bars) within the indicated genotypes. Contingency analysis (Fisher’s exact test) was performed to evaluate statistical differences (g: *n* = 125 WT cells and 140 p73KO cells; g': *n* = 117 WT cells and 127 TAp73KO cells; images from three mice per genotype were analyzed ; ***p < 0.001)

**Fig. 4:**
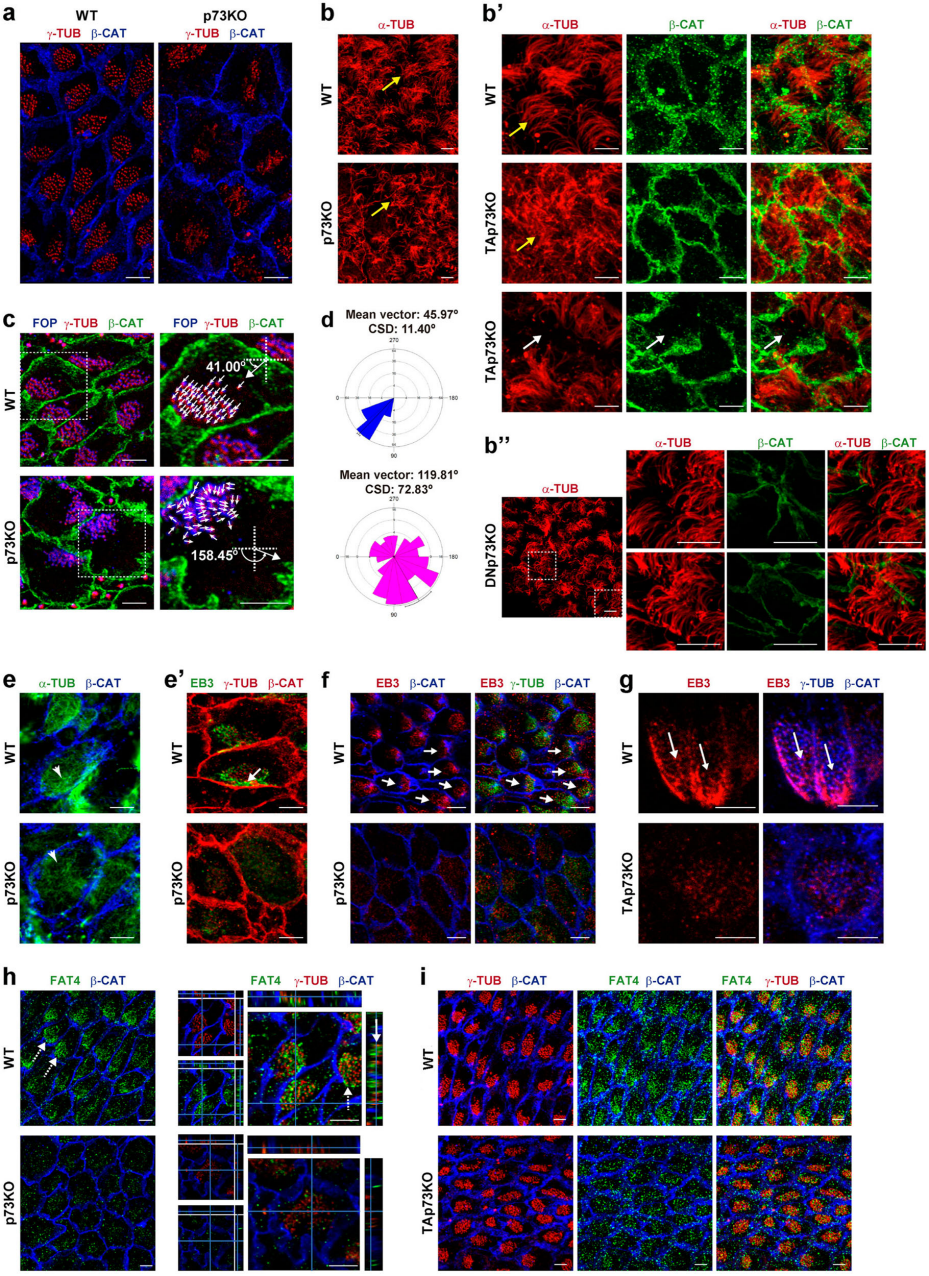
TAp73 regulates cilia organization and polarized junctional MT assembly required for rPCP, and is essential for the subcellular arrangement of the microtubule organizing protein, Fat4. **a**–**i** Representative confocal images of WMs from WT, p73KO (**a**, **b**, **c**–**f**, **h**), TAp73KO (**b′**, **g**, **i**) and DNp73KO (**b″**) mice stained with the indicated antibodies. **b**, **b″** Immunostaining against α-tubulin (red) was used to visualize the cilia axoneme. Scale bar: 10 µm. Parallel and organized bundles of cilia in WT and DNp73, but disorganized in p73KO and TAp73KO-ECs are marked by yellow arrows. White arrows indicate cells lacking cilia axoneme. **c**, **d** To delineate cilia polarity and quantified rPCP, combined staining of FGFR1 Oncogene Partner (FOP, blue) and γ-tubulin (red) on WT and p73KO WMs was performed. Scale bar: 5 µm. The direction of each BBs was drawn by a vector (white arrows) from FOP-dot to γ-tubulin-dot within a BB. An angle of polarity, named rotational angle (RA), was drawn with respect to the horizontal axis for each BB and the mean RA for each cell was determined. **d** Mean vectors and circular standard deviation (CSD) of RAs around the mean in WT and p73KO were calculated (*n* = 105 cells from five mice per genotype). Data were graphically represented using Oriana software and statistically compared by circular analysis (Watson’s U2 test). **e**–**g** Representative confocal images of WMs from the indicated genotype stained for α-tubulin to mark MTs (**e**, green) and for EB3 to determine MT polarization (**e′**, green; **f**, **g**, red). **e** Arrowheads indicate the MT-meshwork underlying BB-clusters. Scale bar: 5 µm. **e′**–**g** White arrows show a second polarized MT network located at anterior intercellular junctions illustrating their coordinated orientation. Scale bar: 5 µm. **h**, **i** WMs from WT and p73KO (**h**) or TAp73KO (**i**) mice were stained against Fat4 (green), γ-tubulin (red), and β-catenin (blue). Scale bar: 5 µm. **h** Fat4 polarized distribution was indicated by dashed arrows. Orthogonal projections show the close association of Fat4 and the BBs indicated by a white arrow

Statistical analysis and Figure generation were performed with GraphPad Prism 7.04 software (Figs. 1–3, 6, 7) and Oriana 4 Software (Fig. 4). Data are expressed as means ± standard error (S.E.) of the mean. Differences were considered significant when *p* < 0.05 (*p < 0.05, **p < 0.01, ***p < 0.001). To test for normal distribution D’Agostino and Pearson tests were performed and non-parametric assumptions were made when necessary. In that case, the means of two experimental groups were compared with Mann-Whitney’s test; otherwise, unpaired two-tailed Student’s *t*-test was applied. In case of multiple comparisons, statistical differences were evaluated using either analysis of variance or Kruskal-Wallis test with Dunn’s multiple comparisons. To compare docking between WT and p73KO-ECs, a contingency analysis (Fisher’s exact test) was performed. Data in Fig. 4 are graphically represented using Oriana software (Kovach Computing Services, Anglesey, Wales) and statically compared by circular analysis (Watson’s U2 test).

**Fig. 5:**
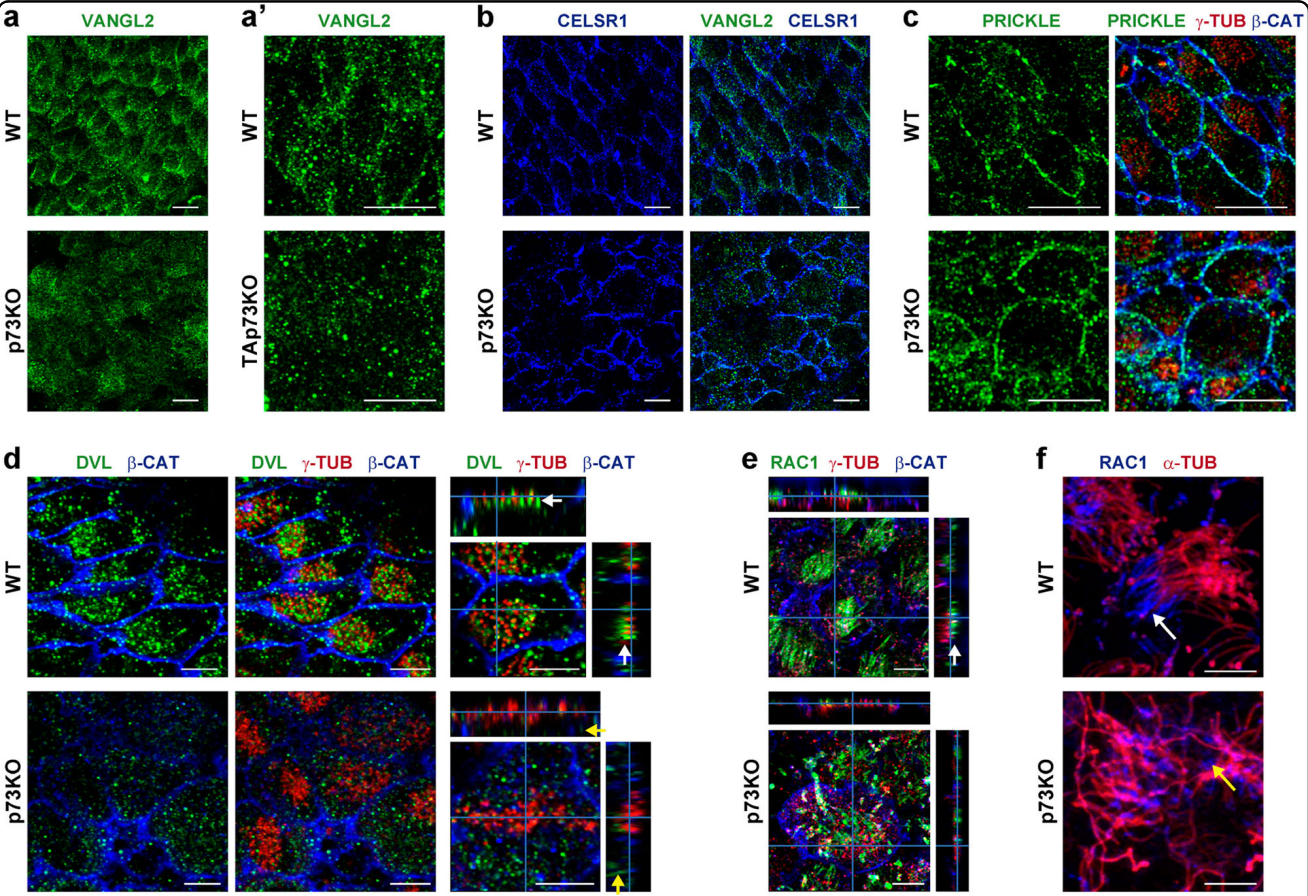
p73 is essential for the polarized junctional assembly of PCP-core proteins and the asymmetric localization of their downstream signaling effectors, Dvl2 and Rac1, in ECs. **a**–**c** WMs from WT, p73KO (**a**), or TAp73KO (**a′**) were stained for PCP-core proteins Vangl2 (**a**, **a′**, **b**, green), Celsr1 (**b**, blue), and Prickle (**c**, green). Scale bar: 10 µm. **d**–**f** ECs were stained with anti-γ-tubulin (**d**, **e**, red) and α-tubulin (**f**, red) to visualize the BBs and axoneme, respectively. PCP-effectors were marked by anti-Dvl2 (**d**, green) and anti-Rac1 (**e**, green; **f**, blue). Orthogonal projections (**d**, **e**) show Dvl2 and Rac1 localization, indicated either by white arrows (WT) or yellow arrows (p73KO). Scale bar: 5 µm

**Fig. 6:**
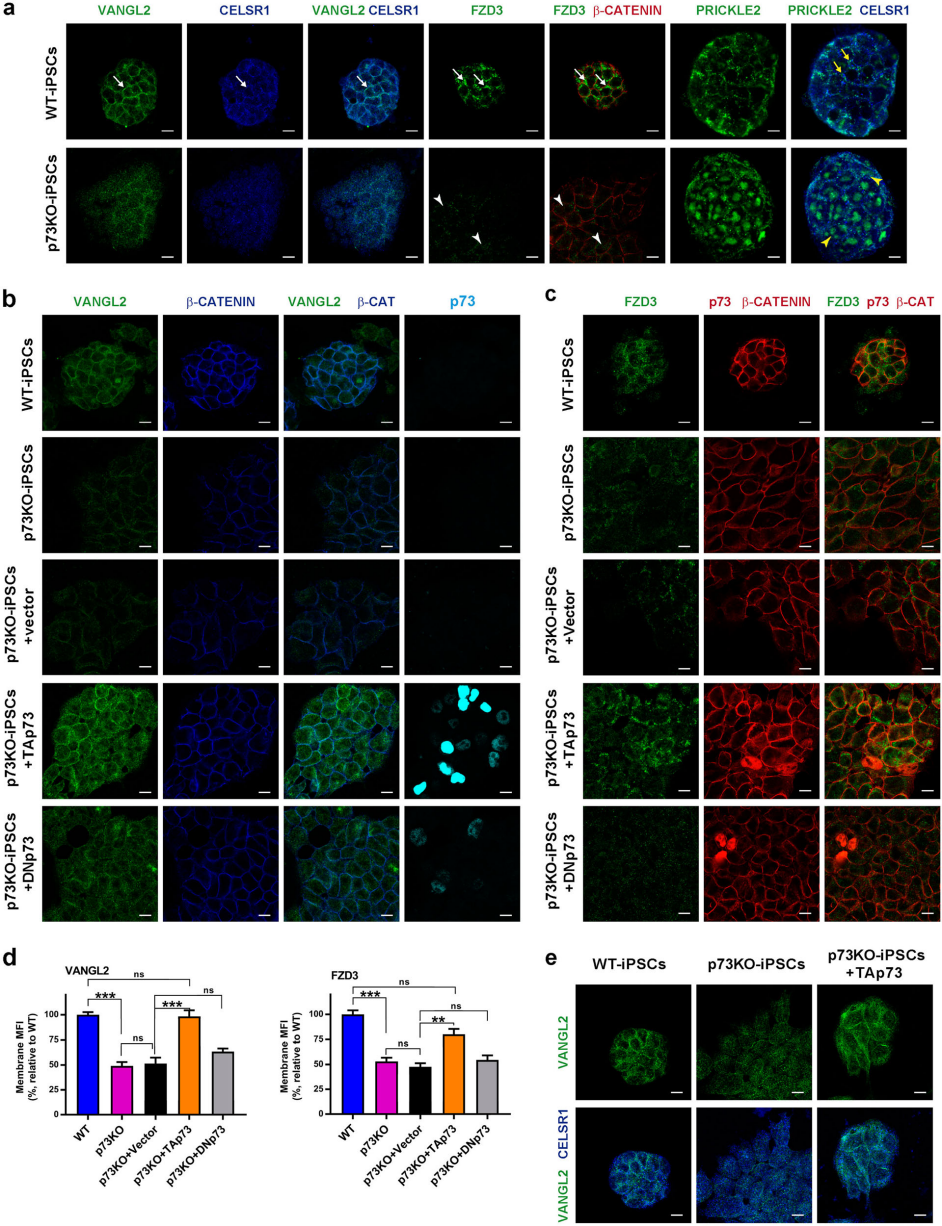
p73 is necessary and sufficient for Vangl2 and Fzd3 membrane localization in vitro. **a**–**e** Representative confocal images of WT and p73KO-iPSC, transfected with the indicated expression vectors (**b**–**e**). PCP-core proteins were visualized by staining with anti-Vangl2 (**a**, **b**, **e**, green), anti-Celsr1 (**a**, **e**, blue), anti-Fzd3 (**a**, **c**, green), and anti-Prickle2 (**a**, green), and plasma membrane by β-catenin (a,c, red; b, blue). **b**, **c** Transfected TAp73 and DNp73 (**b**, light blue; **c**, red) were visualized using specific antibodies. Scale bar: 10 µm. **d** Vangl2 and Fdz3 localization at the plasma membrane was quantified by ImageJ software. Graphs represent the mean florescence intensity (MFI). Two independent transfection experiments were performed with two clones per genotype. Five pictures from each experiment were randomly selected and quantified (*n* = 20 pictures per set). Bars represent mean values ± S.E. Kruskal-Wallis test together with Dunn’s multiple comparisons test was performed to evaluate statistical differences; **p < 0.01, ***p < 0.001, n.s: non-significant. **e** Ectopic expression of TAp73 restores Celsr1 (blue) and Vangl2 (green) colocalization. Scale bar: 10 µm

**Fig. 7:**
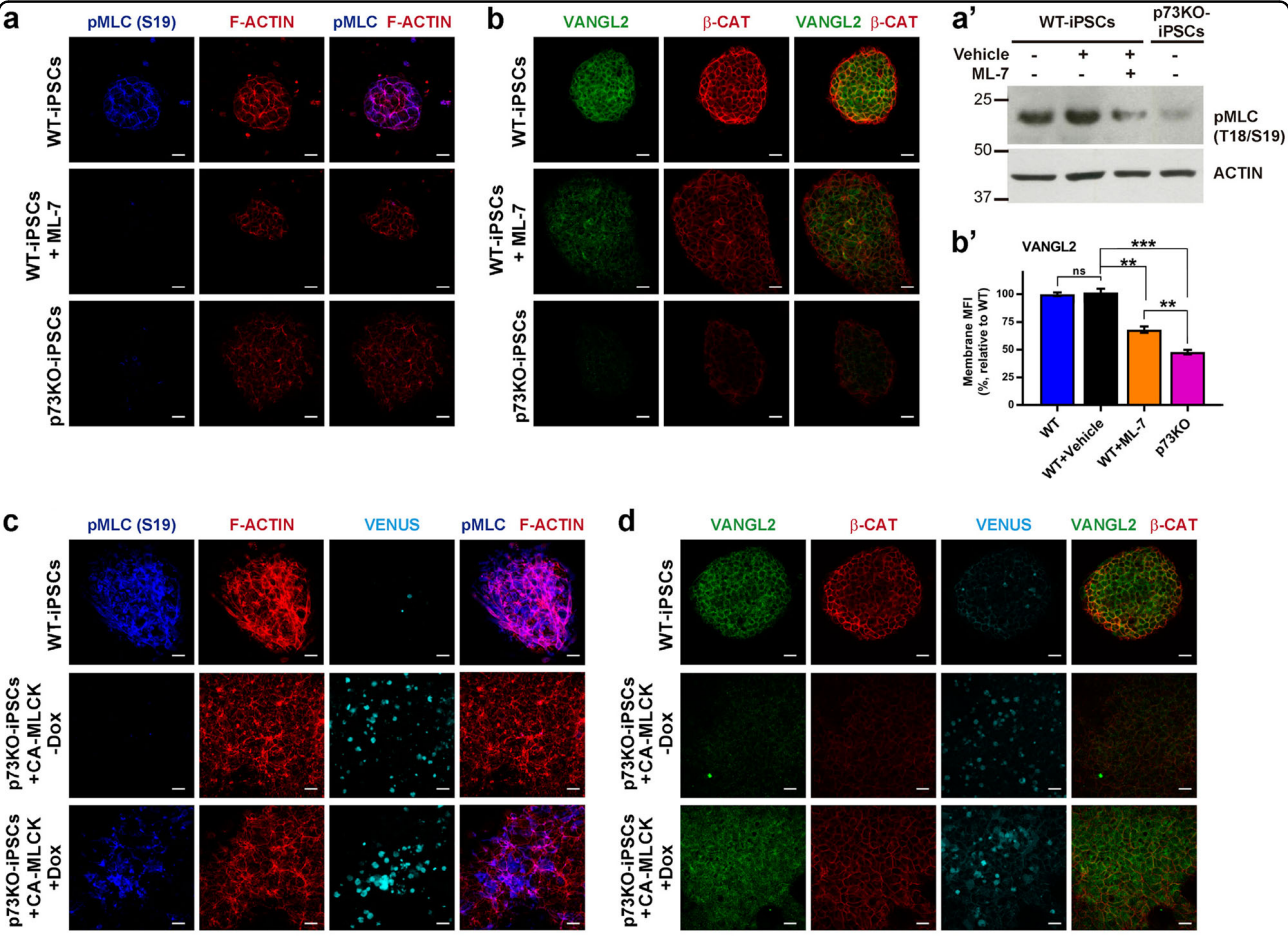
MLCK activity is required, but not sufficient, for PCP-core protein membrane localization **a** WT and p73KO-iPSCs were treated with MLCK inhibitor, ML7, and p-MLC (**a**) or Vangl2 (**b**) localization was analyzed by confocal microscopy. a' (bold font) The effect of ML7 treatment over p-MLC levels was confirmed by western blot **b'** Experiments to quantify Vangl2 localization at the plasma membrane were performed as in Figure 6.b. **c**, **d** p73KO-iPSCs were transfected with inducible constitutively active MLCK (CA-MLCK); VENUS reporter expression shown in light blue) and upon MLCK induction, p-MLC (**c**) and Vangl2 (**d**) colocalization with F-actin at the cellular cortex was analyzed by confocal microscopy. Scale bar: 20 µm

## Results

### p73 coordinates tPCP establishment through the regulation of polarized NMII activity

To address tPCP in transforming RGCs^3^, we quantified primary cilia displacement (CD) from the cell center in WMs from P1 WT and p73KO lateral wall (LW) of the lateral ventricle. (Fig. 1a, white arrows). We found reduced CD in p73KO-RGCs. Thus, in the absence of p73, tPCP establishment at single-cell level is either delayed or defective. We next analyzed the effect of p73 deficiency on NMII activation and localization, both essential for tPCP establishment^15^. In WT-RGCs, activated NMII (p-MLC) was located in distinct puncta at the plasma membrane (Fig. 1b), colocalizing with the primary cilia BB (inserts, white arrows). At the same stage, p73KO-RGCs, with defective CD, have lower levels of p-MLC, especially at the plasma membrane (Fig. 1b), altogether confirming that NMII activation is associated with tPCP establishment.

In immature ECs, BBs packed tightly during migration and get arranged into clusters, termed “crescents”, coordinately displaced toward the anterior apical surface (Fig. 1c, arrows). p-MLC was closely associated to BB within crescents (Fig. 1c, dotted box, Fig. 1d, arrows), advocating the idea that NMII activation plays a fundamental role in BB polarized migration^15^. p73KO-ECs had less CD, crescents were never detected (Fig. 1a, c), and their BBs were scattered throughout the apical surface (Fig. 1c, solid arrows) with little p-MLC associated to them (Fig. 1d, green and red arrows and 1e, arrowheads). p-MLC also localize in puncta at membrane junctions, polarized in the same direction of the BB-cluster (Fig. 1c, dotted box), which are reduced in p73-deficient cells (Fig. 1e), highlighting the correlation between p73 expression and NMII activation and localization.

Analysis of immature ECs from TAp73KO brains, lacking only TAp73 isoforms^31^, revealed a significant decrease in BB-cluster displacement (Fig. 1f) in consonance with their low p-MLC levels (Fig. 1g). Moreover, CD in neighboring TAp73KO-ECs followed different directions, confirming the lack of tissular coordination (Fig. 1f, yellow arrows). Analysis of DNp73KO-ECs, lacking DNp73 isoforms^23^, did not reveal defects in BB-cluster displacement and orientation, or NMII activation and localization (Supplementary 1a,b), indicating that TAp73, but not DNp73, plays a key role in tPCP establishment.

TAp73 could regulate tPCP, at least in part, by controlling NMII activity via the regulatory kinase MLCK^15^. A proximal p53 response element (p53RE) had been previously reported within the human *MLCK* promoter^37^ (Fig. 2a). Moreover, ChIP-seq analysis of TAp73-transfected Saos-2 cells^29^ revealed a TAp73 peak, which could correspond to a putative enhancer element (Fig. 2a, p73RE-enh). We performed luciferase and ChIP assays with the identified p53 and p73REs (Fig. 2b, c). Transcriptional analysis in HCT116 cells with the p53RE-*MLCK-*luc reporter^28^ (Fig. 2b, left panel), and in Saos-2 cells with the p73RE-enh-*MLCK*-luc (right panel), demonstrated that TAp73 can significantly activate both reporters. Under the same conditions, DNp73 had a weak transactivating activity on the proximal p53RE but none over the enhancer. In inducible-TAp73-Saos-2 cells^26^ TAp73 induction correlated with increased MLCK expression (Supplementary 2a) and ChIP assays revealed a significant TAp73-interaction with both sites (Fig. 2c). To demonstrate the relevance of this interaction in a physiological context, we searched for p73/p53REs (JASPAR Core Vertebrata Database) in the murine *Mlck* gene and predicted a strong p73RE within the proximal promoter (−1741 bp from Exon1*^38^, Fig. 2a). ChIP assays showed a significant interaction of endogenous TAp73 with this site in WT, but not in p73KO brain tissue (Fig. 2d). p73-deficiency correlated with lower *Mlck* levels in brain (Fig. 2e and Supplementary 2b), in iPSCs^25^ (Fig. 2f) and in retina and kidney, but not in bladder (Supplementary 2c–e). Moreover, TAp73 overexpression in p73KO-iPSCs significantly induced *Mlck* levels (Fig. 2g, h). These data, as a whole, uncover *MLCK* as a direct TAp73-transcriptional target.

MLCK influences cortical localization of NMII, which functions as a cortical organizer to concentrate E-cadherin to *zonula adherens*^39^. Thus, p73-deficiency might affect the establishment of epithelial cell junctions. To address this, we used iPSCs, a model with epithelial-like cell junctions^25,40^. In WT-iPSCs, p-MLC is strongly expressed at intercellular junctions localizing with F-actin (Fig. 3a, arrows). p73KO-iPSCs, with an impaired epithelial phenotype^25^, had less and mostly cytoplasmic p-MLC (Fig. 3a and Supplementary 3a), not colocalizing with F-actin. TAp73 overexpression induced NMII phosphorylation (Supplementary 3b), restoring p-MLC levels and localization at plasma membrane in p73KO-iPSCs (Fig. 3b, arrows). In agreement, upon TAp73 induction, p-MLC increases and colocalizes with cortical actin bundles^41^ (Supplementary 3c, arrows), highlighting the functional interaction of TAp73, NMII activity, and the actin cytoskeleton.

To address the functional linkage between p73 and the actin cytoskeleton in vivo, we examined F-actin distribution in ECs. These cells displayed cortical F-actin staining^3^ (Fig. 3c, white arrow), a polarized and organized actin apical lattice between the BBs (orthogonal projections, white arrowheads), and a set of sub-apical actin filaments underneath the BBs (blue arrowheads). p-MLC puncta appeared to be associated to the actin lattices within the BBs, as well as with the cortical actin anchored to the plasma membrane (Fig. 3e, white arrowheads and arrows, respectively), suggesting that activated NMII have a key role orienting and keeping together the actin meshwork and, concomitantly, the BBs clusters. Consistent with this hypothesis, p73-deficient cells, with less p-MLC, lacked the organized apical actin lattices associated with BBs and the sub-apical actin filaments (Fig. 3d). Moreover, p-MLC puncta were not associated to the BB cluster or polarized at the plasma membrane (Fig. 3f, yellow arrowheads and arrows, respectively).

Thus, p73-deficiency results in blunted NMII activation leading to a failure in the actin-network assembly, implicated in BB apical migration, docking^42^, and spacing^43^. In agreement, BBs were clearly identified and aligned with the plasma membrane in WT-ECs (Fig. 3g, g'), but p73KO-ECs (Fig. 3g) or TAp73KO-ECs (Fig. 3g′) had BBs scattered and deep within the cytoplasm, demonstrating TAp73 essential role in sub-apical cytoskeleton dynamics and BB docking.

### TAp73 regulates the polarized junctional MT assembly promoting PCP-core complex asymmetric localization

p73 deficiency not only affected BB-cluster displacement, docking, and spacing, but also BB orientation (Fig. 4a). WT-ECs displayed well-organized and straight parallel bundles of cilia, while p73KO (Fig. 4b) and TAp73KO-ECs (Fig. 4b′) had disorganized cilia with a “disheveled” appearance (yellow arrows). Similar cilia alterations have been attributed to BBs’ rotational alignment defects^9,11^. Thus, we measured rotational polarity of individual BBs by calculating an angle of polarity formed by a vector (marking the orientation of each BB) and the horizontal axis^3^ (Fig. 4c). Next, we determined the mean vector for each EC and calculated the CSD (Fig. 4d). This value was significantly larger in p73KO than in WT cells (*p* < 0.001), demonstrating p73 requirement for rPCP. Only few TAp73KO-ECs lacked cilia axoneme (Fig. 4b′, white arrows), but we did not detect ciliary axoneme defects in DNp73KO-ECs, which displayed well-organized and parallel bundles of cilia and well-oriented BBs (Fig. 4b″ and Supplementary 1b).

p73 regulation of actin dynamics, by itself, could not fully explain the generation of refined rPCP^40^. Thus, we expanded our analysis to the MT cytoskeleton, since polarized MT dynamics is fundamental for rPCP^3,12,13^. α-tubulin staining revealed a fine and polarized MT-meshwork (Fig. 4e, arrowhead), with similar disposition to the actin lattice within BB (Fig. 3c). In p73KO-ECs this meshwork was lax and disorganized (Fig. 4e, arrowhead). To address MT-growth orientation, we analyzed EB3, a plus-end MT protein, which marks newly synthesized MTs. In WT cells, a second MT network grew asymmetrically from the center of the cell toward the anterior region of the apical cell cortex contacting the plasma membrane (Fig. 4e′, arrow). These MT-anchoring points^3,12,44^ were polarized at tissue-level (Fig. 4f, g, arrows). Interestingly, p73 deficiency resulted in lower and diffuse levels of EB3 staining around the scattered BBs, and a total absence of polarized MT-anchoring points at cell junctions (Fig. 4e–g), demonstrating TAp73 requirement for polarized MT network formation in developing ECs.

The protocadherins Fat (Fat4 in mammals^4^) and its ligand Dachsous (Ds) conform the global-PCP module, known to control the polarized junctional alignment and asymmetry of MT^13,45^. Confocal images of WT-ECs revealed Fat4 polarized distribution toward the anterior apical cell surface (Fig. 4h, dashed arrows). Orthogonal planes revealed that Fat4 was closely associated with the BBs (white arrow). p73KO-ECs had weak Fat4 staining, scattered throughout the cytoplasm without association with the BBs, lacking any asymmetric distribution (Fig. 4h, i).

Polarized MTs are required for targeting PCP proteins to the proper asymmetric membrane domains^12,46^. PCP-core molecular complexes formed by Vangl2, Celsr1, and Pk2 displayed asymmetric distribution in WT-ECs (Fig. 5a–c), with Vangl2 exhibiting the characteristic zigzag pattern^3^ indicative of tissular coordination of polarity. This asymmetric distribution was completely lost in p73KO-ECs (Fig. 5a–c) and Vangl2 had cytoplasmic localization (Fig. 5a, a′) suggesting a defect in Vangl2-vesicle trafficking.

PCP-core proteins transduce the polarity signals through the adaptor protein Dvl2^9,15^ and its effector Rac1^47^. Orthogonal projections demonstrated that Dvl2 was located at the base of the individual BBs (Fig. 5d, white arrows) clustered at the anterior cell surface, establishing a polarized Dvl2-distribution. As expected, Rac1 was localized on top of the BBs (Fig. 5e, white arrows) at the base of the axoneme (Fig. 5f, white arrows). In p73 absence, Dvl2 did not associate with BBs (Fig. 5d, yellow arrows), and Rac1 was found scattered throughout the disheveled axonemes (Fig. 5e, f, yellow arrow), all in consonance with a lack of the polarized anchoring structures required to scaffold and orient the PCP signaling machinery.

### TAp73 induces PCP-core protein membrane localization and modulates MT dynamics

TAp73 acts as a transcriptional integrator of multiciliogenesis^18,19^ and regulator of EC differentiation^17^. Therefore, it was possible that like in airway epithelial cells^16^, the defects observed in p73KO-ECs were a consequence of their defective differentiation and ciliogenesis impairment. To rule this out, we analyzed PCP-core protein localization in a ciliogenesis-independent model, the iPSCs. In WT-iPSCs, Vangl2, Celsr1, and Fzd3 localized at the membrane, with intense staining at the lateral junctions (Fig. 6a, white arrows). Prickle2 also had cortical localization, close to Celsr1 (Fig. 6a, yellow arrows). Lack of p73 resulted in cytoplasmic Vangl2, which had lost most of its colocalization with Celsr1, confirming p73 requirement for Vangl2 membrane localization. In p73KO-iPSCs Fzd3 had faint and diffuse cytoplasmic staining (Fig. 6a, arrowheads), while little Prickle2 localized at the cell cortex associated with Celsr1 (Fig. 6a, yellow arrowheads). To test whether p73 was sufficient for PCP-core protein membrane localization, we expressed TAp73 or DNp73 in p73KO-iPSCs and analyzed the restoration of Vangl2 and Fzd3 at the plasma membrane and Celsr1 colocalization. Confocal microscopy (Fig. 6b, c, e) and image quantification (Fig. 6d) revealed that TAp73, but not DNp73, could rescue the p73KO phenotype and demonstrated that TAp73 is necessary and sufficient for membrane localization of PCP-core proteins, independently of its function as multiciliogenesis regulator.

MLCK is required for the establishment of epithelial cell junctions, which are necessary for polarized transport. Moreover, in some systems MLCK mediates intracellular trafficking over MTs^48^. Therefore, we asked whether MLCK could be involved in PCP-core protein membrane transport. We treated iPSCs with the MLCK inhibitor ML7^15^ and confirmed that upon treatment p-MLC went down, disappearing from intercellular junctions (Fig. 7a, a′). Concomitantly, Vangl2 membrane localization decreased (Fig. 7b, b′), partially phenocopying p73KO-iPSC. Furthermore, overexpression of constitutively active MLCK (CA-MLCK^49^) in p73KO-iPSCs resulted in an enhancement of p-MLC levels (Fig. 7c) and a partial restoration of PCP-core proteins membrane localization (Fig. 7d), indicating that MLCK/NMII signaling pathway is required for PCP-core proteins membrane delivery, but other factors are also involved.

**Fig. 8:**
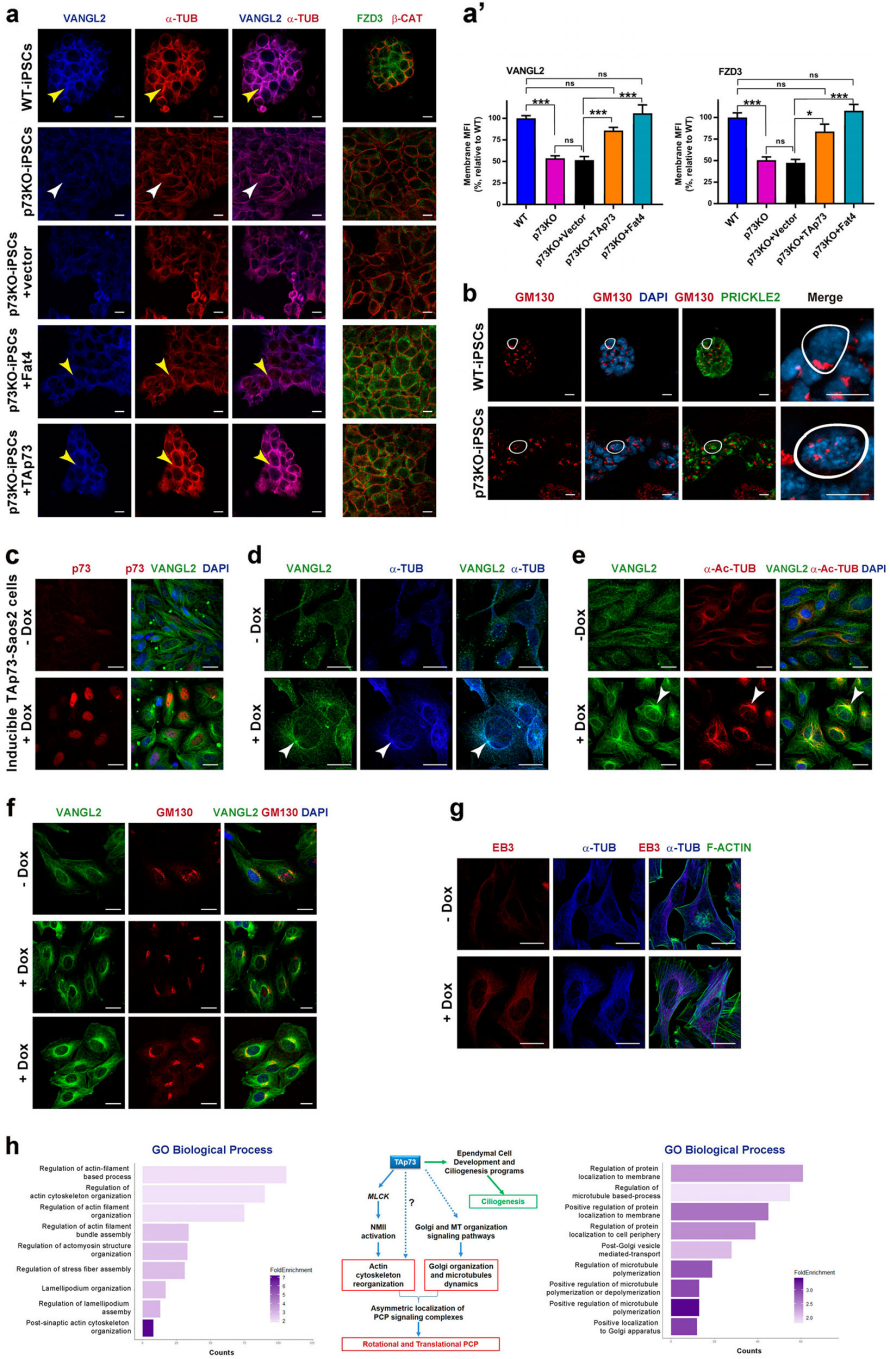
TAp73 modulate MT dynamics and actin cytoskeleton. **a** Analysis of Vangl2 and Fzd3 membrane localization by confocal microscopy on WT and p73KO-iPSC, transfected with the indicated expression vector. Plasma membrane was visualized by β-catenin staining (red), MT by α-tubulin (red), Vangl2 (blue), and Fzd3 (green). The lax meshwork of MTs in p73KO is marked by white arrowheads while tight cortical networks are marked by yellow arrowheads. **a′** ImageJ quantification of the amount of Vangl2 and Fdz3 localized at the plasma membrane. Graphs represent the mean florescence intensity (MFI). Two independent transfection experiments were performed with two clones per genotype. Five pictures from each experiment were randomly selected and quantified (*n* = 20 pictures per set). Bars represent mean values ± S.E. Kruskal-Wallis test together with Dunn’s multiple comparisons test was performed to evaluate statistical differences; *p < 0.05, ***p < 0.001, n.s: non-significant. **b** The Golgi apparatus was marked by GM130 (red) and in the magnification panel the contour of a single cell is delineated by a white line. **c**–**g** Analysis of MT cytoskeleton changes induced upon TAp73 expression (+Dox) in TAp73-Saos-2 cells stained with specific antibodies against TAp73 (**c**, red), Vangl2 (**d**–**f**, green), α-tubulin (**d**, **g**, blue), acetylated α-tubulin (**e**, red), EB3 (**g**, red), and Phalloidin (**g**, green). Nuclei are visualized by Dapi (**c**, **e**, **f**). **a**, **b** Scale bar: 10 µm; **c**–**f** Scale bar: 20 µm. **h** Plot representing GO PANTHER enrichment analysis of TAp73 peak-containing differentially expressed genes from Saos-2 cells with ectopic expression of TAp73 (GSE15780). GO Biological process complete was used as annotation data set. Proposed model of TAp73 regulation of PCP in ependymal cells through the regulation of microtubules and actin organization signaling pathways

To determine if a defect in MT dynamics was causing the observed p73KO-iPSCs defects, we asked whether Fat4, which promotes the formation of polarized MT networks, could rescue the phenotype, similarly to TAp73 overexpression^13,50^. p73KO cells displayed a loosely formed MT meshwork (Fig. 8a, white arrowheads); however, upon Fat4 or TAp73 expression, the MT cytoskeleton formed a tight cortical network at the proximity of the cell periphery (where PCP-core proteins were localized), resembling WT phenotype (Fig. 8a, yellow arrowheads). Concurrently, Vangl2 and Fzd3 levels at the plasma membrane significantly increased, approaching those of WT-iPSCs (Fig. 8a′). Thus, Fat4 was sufficient to rescue the MT disposition and restore PCP-core proteins membrane localization in p73KO-iPSCs, confirming that defective MT dynamics were an underlying cause of p73KO phenotype.

MTs play a central role in the Golgi complex integrity and agents that alter MT distribution induce Golgi fragmentation^51,52^. As expected, *cis*-Golgi fragmentation was detected in p73KO-iPSCs (Fig. 8b). Next, we analyzed MT cytoskeleton and Vangl2 localization in inducible-TAp73-Saos-2 cells. Untreated cells, with very low levels of p73 (Fig. 8c), displayed a lax MT disposition and diffuse cytoplasmic Vangl2 staining (Fig. 8c–e). Upon TAp73 induction, changes in Vangl2 expression pattern, concurrently with changes in MTs disposition, were observed. These adopted a radial structure that radiated from a juxtanuclear cellular compartment where Vangl2 was enriched (Fig. 8d, arrowheads). This subcellular compartment accumulated acetylated-tubulin, which colocalized with Vangl2 (Fig. 8e, arrowheads) and Golgi marker GM130 (Fig. 8f). Therefore, it was consistent with Golgi-derived MTs, reported to preferentially accumulate acetylated-tubulin^53^ and indispensable for cell polarity^54^. Upon TAp73 induction we detected an increase on EB3 levels (Fig. 8g), confirmed by RT-PCR in inducible-TAp73-Saos-2 cells and p73KO-iPSCs (Supplementary Fig. 4a, b), all demonstrating that p73 could induce changes in MT dynamics. These changes were accompanied by a dramatic alteration in actin cytoskeleton and peripheral stress fiber architecture, readouts of MLCK enhancement^55^.

To determine whether TAp73 could induce transcriptional programs involved in these processes, we analyzed previously published TAp73β-ChIP-seq/RNA-seq data from Saos-2 cells (GSE15780)^29^. We used differentially expressed genes (DEGs) upon TAp73β expression that contained TAp73 peaks either at the promoter and/or at regulatory regions (within 25 kb). GO analysis identified terms like “Regulation of MT based processes”, “Regulation of MT polymerization”, “Post-Golgi vesicle–mediated transport”, and “Regulation of Actin cytoskeleton organization” as highly enriched biological processes (Fig. 8h and Supplementary 4c,d). These DEGs included key regulators of Golgi organization and MT dynamics like *AKAP9* (*AKAP 450*), *PDE4DIP*, *GOLGA1*, or *CLIP1* also required for cell polarity^56,57^. Among actin cytoskeleton regulators, we confirmed *MYLK* (*MLCK*) as a TAp73β-DEG. These results support that TAp73 modulates, directly and/or indirectly, transcriptional programs regulating actin and MT dynamics and Golgi organization.

## Discussion

Polarity establishment relies on polarized actin and MT cytoskeletal rearrangements, but knowledge of the signals that organize these processes remains incomplete^43^. This work demonstrates an essential role of TAp73 in PCP through the regulation of both, actin and MT-polarized network assembly, providing a model to the mechanism of planar polarization regulated by TAp73 (Fig. 8h).

We show that TAp73, but not DNp73, is necessary for the efficient tPCP establishment in RGCs, suggesting that the initial mechanoreceptors signaling, which instruct primary CD, cannot work properly in the absence of p73. While TAp73KO-ECs show PCP- and BB-docking defects, cilia axoneme alterations are milder than in the total p73KO mice, suggesting that DNp73 could have a compensatory role. However, consistent with the lack of DNp73 expression in ECs^23^, we did not detect any cilia or PCP defects in DNp73KO-ECs. These data suggest that compensatory and redundant ciliary programs are induced in the absence of TAp73 when DNp73 is present, but not in total p73 deficiency. Nevertheless, these signaling programs do not compensate for PCP establishment in the absence of TAp73, uncoupling ciliogenesis, and PCP signaling in ECs.

Actin remodeling and the actin-based motor protein NMII are essential to establish tPCP^15^. Our results support a model in which NMII activation and membrane localization correlates with anterior migration of the BB crescent. Mechanistically, we identify TAp73 isoform as an upstream modulator of NMII through the transcriptional regulation of MLCK in vivo and in vitro. This directly links p73 with actin microfilament dynamics, essential for proper cilia docking and spacing, and for the establishment of global coordination of cilia polarity^43,58^.

PCP regulation in epithelial cells relies on the PCP-core and the Fat/Ds modules to generate morphological asymmetries^59^. PCP-core complexes transduce polarity signals through the adaptor protein Dvl2^9,15^ and its effectors aPKC, Rac1, and RhoA, leading to reorganization of the actin cytoskeleton^9^. We show that Rac1 is localized at the base of the axoneme in a region corresponding to the ciliary pocket, a proposed interface with the actin cytoskeleton and a platform for vesicle trafficking^60^. These molecular complexes are not established in the absence of p73. We report for the first time in ECs^4^, the expression of the Fat/Ds-module components^13,45^ at the base of the cilia closely associated with the BBs, supporting its role in ciliary functions. Interestingly, p73-deficient cells displayed a defective global-PCP signaling that could be underlying, at least in part, the lack of polarized MT-junctional structures in p73KO cells.

Polarized MTs are an integral component of early steps in cell polarity driving asymmetric accumulation of PCP-core proteins^45,61^. The cytoplasmic distribution of Vangl2 in p73-deficient ECs suggests a possible defect in Vangl2-vesicle trafficking and points toward a possible role of p73 in MT dynamics. Moreover, disassembling of MTs by nocodazole treatment of brain ventricles impairs the trafficking and distribution of PCP-core proteins in ECs^3^, phenocopying the observed p73KO phenotype. Lack of p73 affected both sets of MT described in ECs. Such defects could impair PCP-core proteins traffic to the plasma membrane at the MT-anchoring points and the assembly of PCP-asymmetric complexes.

We demonstrate that TAp73 is necessary and sufficient for membrane localization of PCP-core proteins independently of multiciliogenesis. Moreover, MLCK/NMII signaling pathway is necessary for PCP protein plasma membrane delivery, but not sufficient to completely rescue p73KO phenotype. Since the reciprocal interaction between MTs and the actin cytoskeleton is necessary for intracellular transport^62^, it is possible that similar functional interactions are triggered by TAp73 in this system.

We found that p73-deficient ECs displayed lower levels of EB3, a TIP+-binding protein required to modulate MT dynamics and the Golgi apparatus organization^63^. Interestingly, EB1/3 disruption is known to perturb non-centrosomal MT organization^57^. Congruently, TAp73 overexpression induces enhanced EB3 staining and dramatic changes in the dynamics of non-centrosomal Golgi-derived MTs, indispensable for post-Golgi trafficking and cell polarity^54^.

GO analysis of DEGs containing TAp73 peaks showed enrichment in Golgi organization and MT dynamics, as well as actin cytoskeleton organization GO-terms, confirming TAp73 role as modulator of actin and MT dynamics signaling programs. However, further studies will be required to unravel how p73, directly or indirectly, regulates these modules.

Altogether, our data demonstrate that TAp73 not only regulates ECs development and ciliogenesis but also is an essential regulator of PCP establishment. This novel p73 function as an epithelial architect is not only fundamental during development but could also play a relevant role in TAp73 tumor suppression.

## Electronic supplementary material

Supplementary Figure legends

Supplementary Information

Supplementary Figure 1. Lack of DNp73 has no effect on PCP establishment or pMLC localization or rotational PCP Basal Bodies organization

Supplementary Figure 2. p73 expression correlates with MLCK levels

Supplementary Figure 3. TAp73 overexpression induces NMII activation

Supplementary Figure 4. TAp73 overexpression results in changes in “Golgi and MT organization” signaling pathways as well as Actin dynamics
