# Supplementary material for: p73 regulates ependymal planar cell polarity by modulating actin and microtubule cytoskeleton
Source: Cell Death Dis. 2018 Dec 5;9(12):1183. doi: 10.1038/s41419-018-1205-6 (PMC6281643; doi:10.1038/s41419-018-1205-6)
Supplement: Supplementary file 1 — Supplementary Figure legends [file 41419_2018_1205_MOESM1_ESM.docx]

**Supplementary Figure legends**

**Supplementary Figure 1. Lack of DNp73 has no effect on PCP establishment or pMLC** localization or rotational PCP Basal Bodies organization

(a) Representative confocal images of Whole Mounts (WMs) from WT and DNp73 mice stained by anti-pMLC (green), anti-γ-tubulin (red) and β-catenin (blue) staining. (b) Combined staining marking FGFR1 Oncogene Partner (FOP, blue), which localized at the base of cilia, and γ-tubulin (red) for the basal feet, to determine BB organization within the cells and cilia polarity. The membrane is delineated by β-catenin (blue) staining.

**Supplementary Figure 2. p73 expression correlates with MLCK levels**

(**a**) Analysis of TAp73 and Mlck induction by qRT-PCR (upper panel) or western blot analysis (lower panel) in TAp73-SaOs2 cells upon TAp73 induction by Doxycycline treatment. Three independent transfection experiments were performed with two clones per genotype. qRT-PCR assays were repeated four times by duplicate. (**b-e**) Analysis by qRT-PCR of *Mlck* expression in different tissues: Brain (**b**); Retina (**c**); Kidney (**d**) and Bladder (**e**) from WT and p73KO mice. Bars represent mean values ± S.E. *p<0.05, **p<0.01, ***p<0.001.

**Supplementary Figure 3. TAp73 overexpression induces NMII activation**

(**a-b**) Western blot analysis of NMII activation (p-MLC) in WT vs. p73KO-iPSC (**a**) or in TAp73-SaOs2 cells (**b**) after TAp73 induction by Doxycycline treatment. (**c**) Detection of activated NMII and actin cytoskeleton by p-MLC and phalloidin staining respectively, in TAp73-Saos2 cells with arrows indicating p-MLC colocalization with cortical actin bundles. Scale bar: 10 µm.

**Supplementary Figure 4. TAp73 overexpression results in changes in “Golgi and MT organization” signaling pathways as well as Actin dynamics**

**(a-b)** RT-PCR analysis of EB3 (*MAPRE*3) expression levels in inducible TAp73-Saos2 (**a**) cells and p73KO-iPSCs (**b**) upon TAp73 overexpression. Bars represent mean values ± S.E. *p<0.05, **p<0.01, ***p<0.001. (**c**) Gene Onthology term analysis using selected TAp73-peak containing DEGs after ectopic expression of TAp73 from GSE15780, using DAVID(c) and PANTHER (d) FUNCTIONAL ANNOTATIONS, showed enrichment in Golgi organization and MT dynamics, as well as Actin cytoskeleton organization. Level of enrichment and FDR values and highlighted genes are indicated in the table.
