# Supplementary material for: p73 regulates ependymal planar cell polarity by modulating actin and microtubule cytoskeleton
Source: Cell Death Dis. 2018 Dec 5;9(12):1183. doi: 10.1038/s41419-018-1205-6 (PMC6281643; doi:10.1038/s41419-018-1205-6)
Supplement: Supplementary file 2 — Supplementary Information [file 41419_2018_1205_MOESM2_ESM.docx]

**SUPPLEMENTAL INFORMATION**

**SUPPLEMENTARY EXPERIMENTAL PROCEDURES**

**Immunostaining of wholemounts (WMs)**

WMs were transferred ventricle side up to a 48-well plate and fixed with 4% paraformaldehyde (PFA) or with ice-cold methanol, depending on the antibody requirement.

Samples fixed at 4% PFA o/n at 4ºC were blocked for 1 hour at RT in blocking buffer (0.1M PBS/10% NDS/0.5% Tx100) and then, incubated for 24 h at 4°C with the indicated primary antibodies (Table S1) diluted in blocking buffer. After that WMs were washed three times during 20 min with 0.1M PBS/0.1% Tx100 and incubated 24 h at 4ºC with secondary antibodies (Table S1).

Samples fixed at 4%PFA 10 min/30 min RT or with methanol 15 min at -20ºC were incubated for 30 min at RT in blocking buffer (0.1M PBS/10% NDS/0.1% Tx100). Then, they were incubated for 90 min at RT with the indicated primary antibodies (Table S1). WMs were washed three times during 5 min with 0.1M PBS/0.1% Tx100 and incubated 1 hour at RT with secondary antibodies (Table S1). DAPI (1µg/mL) was used for nuclear counterstaining and Fluoromount-G (Electron Microscopy Sciences, Hatfield, UK) as mounting media. Samples were analyzed using the Zeiss LSM800 and Olympus FluoView FV10i confocal laser scanning microscopes. Confocal Z-stack images were taken in all cases. Images were processed using ZEN blue software, FV10-ASW 2.1 viewer software and ImageJ software.

**Immunostaining of cell cultures**

For immunofluorescence staining of non-transfected iPSCs, 5·10^4^ cell/cm^2^ were seeded on coverslips in 24-well plates and fixed with 3.7% PFA during 15 min at RT 24h after seeding. Transfected iPSCs and inducible TAp73-Saos-2-Tet-On cells were fixed with 3.7% PFA or 3.7% PFA + 0.5% Tx-100, depending on the antibody requirement, incubated for 15 min in permeabilization buffer (0.1M PBS/0.5% Tx100) and for 1h in blocking solution (0.1M PBS/10% NDS). Cell were stained incubating for 1h at RT with the primary antibodies (Supplementary Table 1) and then, 45 min at RT with the secondary antibodies (Table S1). DAPI (1µg/mL) was used for nuclear counterstaining. FluoromountG (Electron Microscopy Sciences, Hatfield, UK) was used as mounting media.

**Chromatin Immunoprecipitation assay**

ChIP analysis was carried out as previously described^1^ using inducible TAp73-Saos-2-Tet-On cell pellets (20x10^6^ cells) or WT and p73KO brains. Samples were fixed with 1% formaldehyde for 10 min at RT. The reaction was stopped by addition of 0.125 mM glycine for 10 min at RT and cells were washed with PBS and lysed in 0.7% SDS lysis buffer. Cross-linked chromatin was fragmented by sonication to an average size of 400 bp using a Bioruptor® sonicator (Diagenode, Seraing, Belgium). Chromatin was immunoprecipitated with the following antibodies: anti-HA (Y-11) (Santa Cruz Biotechnology, TX, USA), anti-p73 (Abcam, Cambridge, UK) and anti-p53 (Santa Cruz Biotechnology, TX, USA). Antibodies and cell lysates were incubated o/n at 4°C prior to the addition of protein G-coupled magnetic beads (Dynabeads, Invitrogen, Carlsbad, CA, USA) for 4h at 4°C. Negative controls were prepared by incubating parallel samples with non-immune rabbit IgG (Abcam, Cambridge, UK).

The protein-DNA cross-links were reversed in elution buffer (1% SDS and 50 mM Tris-HCl), followed by RNAse treatment overnight at 65°C. The eluted material was incubated with proteinase K for 3 h at 45°C, and the DNA was purified using the QIAquick PCR purification kit (Qiagen, Hilden, Germany). Real time qPCR was performed using FastStart Universal SYBR Green Master (Roche, Basel, Switzerland) in a StepOnePlus Real-Time PCR System (Applied Biosystems, Carlsbad, CA, USA). The signals were normalized to the input (non-immune rabbit IgG immunoprecipitation). Primers encompassing the p53RE and p73RE in the human *MLCK* gene and the p73RE in mouse *Mlck* gene were as follows:

p53RE in human *MLCK* gene. Amplicon, 192bp. (Figure 2B, 2C, left panels).

5′-CATGTCACCGAAGCCAGATC-3’ and 5′-GACAGACGCAGAACCATGAC-3’

p73RE in human *MLCK* gene. Amplicon, 83bp. (Figure 2B, 2C, right panels).

5′-GAGTTGTGGTTTGCCGTGTT-3’ and 5′-AGCACTTTGGGAGGCCAAG-3’

p73RE in mouse *Mlck* gene. Amplicon, 169bp. (Figure 2D).

5′-TTGAGTGTCCAGTGGTGCTC-3’ and 5′-ACACTTGGGAGCAACCAAAA-3’

**Protein isolation and Western Blot analysis**.

Protein extraction and immunoblot was performed as previously described^2^. Membranes were incubated overnight at 4ºC with the following primary antibodies: rabbit anti-pMLC (T18/S19) 1:1000 (Cell Signaling, MA, USA), rabbit anti-pMLC (S19) 1:1000 (Cell Signaling, MA, USA), rabbit anti-MLCK 1:2500 (Abcam, Cambridge, UK), rabbit anti-HA (Y11) 1:1000 (Santa Cruz Biotechnology, TX, USA) and rabbit anti-Actin 1:10.000 (Sigma, MO,USA) followed by the appropriate HRP-conjugated secondary antibodies (Pierce, MA, USA). The enhanced chemiluminescent was detected with Super Signal West-Pico Chemiluminescent Substrate (Pierce, MA, USA). For p-MLC detection cells were lysed in 2× Laemmli sample buffer containing 0.2% β-mercaptoethanol, protease inhibitor cocktail (Merck, Darmstadt, Germany) and phosphatase inhibitor cocktail (Roche, Basel, Switzerland).

1. Martin-Lopez M, Maeso-Alonso L, Fuertes-Alvarez S, Balboa D, Rodriguez-Cortez V, Weltner J*, et al.* p73 is required for appropriate BMP-induced mesenchymal-to-epithelial transition during somatic cell reprogramming. *Cell death & disease* 2017, **8**(9)**:** e3034.

2. Fernandez-Garcia B, Vaque JP, Herreros-Villanueva M, Marques-Garcia F, Castrillo F, Fernandez-Medarde A*, et al.* p73 cooperates with Ras in the activation of MAP kinase signaling cascade. *Cell death and differentiation* 2007, **14**(2)**:** 254-265.

**Table S1. Primary and secondary antibodies and phalloidin staining**

| **Primary antibody** | **Host** | **Dilution** | **Manufacturer** | **Catalog number** | **Fixation requirements in WM IF staining** |
| --- | --- | --- | --- | --- | --- |
| βCatenin | mouse | 1/200 | BD transduction lab. | 610153 | All fixative conditions used in this work |
| βCatenin | rabbit | 1/200 | Cell signaling | 9587 | All fixative conditions used in this work |
| Celsr1 | guinea pig | 1/200 | Gift from Dr. Tissir (University of Louvain, Brussels, Belgium) |  | 4% PFA 30 min RT |
| Dvl2 (H-75) | rabbit | 1/200 | Santa Cruz Biotechnology | sc-13974 | Frozen methanol 15 min -20ºC |
| EB3 (MAPRE3) | rat | 1/200 | AbCam | ab53360 | 4% PFA o/n 4ºC |
| Fat4 | rabbit | 1/200 | Novus Biologicals | NBP1-78381 | Frozen methanol 15 min -20ºC |
| Frizzled3 | rabbit | 1/200 | Sigma-Aldrich | SAB4503170 |  |
| Frizzled3 | goat | 1/500 | R&D Systems | AF1001 |  |
| FOP | mouse | 1/200 | Abnova | H00011116-M01 | 4% PFA o/n 4ºC  Frozen methanol 15 min -20ºC |
| HA (F-7) | mouse | 1/200 | Santa Cruz Biotechnology | sc-7392 |  |
| HA (Y-11) | rabbit | 1/200 | Santa Cruz Biotechnology | sc-805 |  |
| Prickle2 | rabbit | 1/200 | OriGene | TA320126 | Frozen methanol 15 min -20ºC |
| p-MLC2 T18/S19 | rabbit | 1/200 | Cell signaling | 3674 | 4% PFA o/n 4ºC |
| pMLC2 S19 | Rabbit | 1/50 | Cell signaling | 3671-S | 3.7% PFA+0.5%Tx-100 |
| p73 Ab-4 | mouse | 1/200 | NeoMarkers | M-764-P |  |
| Rac1 (23A8) | mouse | 1/200 | Merck-Millipore | 05-389 | 4% PFA o/n 4ºC |
| αTubulin | mouse | 1/200 | AbCam | ab7291 | 4% PFA o/n 4ºC (cilia staining)  4% PFA 10 min RT (MT staining) |
| Tubulin Acetilated | mouse | 1/200 | Sigma-Aldrich | T6793 | 4% PFA o/n 4ºC |
| Tubulin Acetilated | mouse | 1/200 | Abcam | ab24610 | 4% PFA o/n 4ºC |
| γTubulina (C20) | goat | 1/200 | Santa Cruz Biotechnology | sc-7396 | 4% PFA o/n 4ºC  Frozen methanol 15 min -20ºC |
| γTubulina (GTU-88) | mouse | 1/500 | Sigma-Aldrich | T6557 | Frozen methanol 15 min -20ºC |
| Vangl2 (H-55) | rabbit | 1/200 | Santa Cruz Biotechnology | sc67136 | 4% PFA o/n 4ºC |

| **Secondary Antibody** | **Host and Reactivity** | **Dilution** | **Manufacturer** | **Catalog Number** |
| --- | --- | --- | --- | --- |
| Alexa Fluor 488 | donkey anti mouse | 1/1000 | Molecular Probes | A21202 |
| Alexa Fluor 488 | donkey anti rabbit | 1/1000 | Molecular Probes | A21206 |
| Alexa Fluor 488 | goat anti mouse IgG2a | 1/1000 | Molecular Probes | A21131 |
| Cy^TM^3-conjugated | donkey anti mouse | 1/1000 | Jackson ImmunoResearch | 715-165-150 |
| Cy^TM^3-conjugated | donkey anti rabbit | 1/1000 | Jackson ImmunoResearch | 711-165-152 |
| Cy^TM^3-conjugated | donkey anti goat | 1/1000 | Jackson ImmunoResearch | 705-165-147 |
| Alexa Fluor 546 | donkey anti mouse | 1/1000 | Molecular Probes | A10036 |
| Alexa Fluor 568 | donkey anti goat | 1/1000 | Molecular Probes | A11057 |
| Alexa Fluor 594 | donkey anti rat | 1/1000 | Jackson ImmunoResearch | 712-585-150 |
| Alexa Fluor 594 | goat anti mouse IgG1 | 1/1000 | Molecular Probes | A21125 |
| Alexa Fluor 594 | goat anti rabbit | 1/1000 | Molecular Probes | A11012 |
| Alexa Fluor 647 | donkey anti mouse | 1/1000 | Molecular Probes | A31571 |
| Alexa Fluor 647 | donkey anti goat | 1/1000 | Molecular Probes | A21447 |
| Alexa Fluor 647 | goat anti guinea pig | 1/1000 | Molecular Probes | A21450 |
| Alexa Fluor 647 | goat anti mouse IgG2b | 1/1000 | Molecular Probes | A21242 |
| Alexa Fluor 647 | goat anti rabbit | 1/1000 | Molecular Probes | A21244 |

| **Phalloidin Staining** | **Dilution** | **Manufacturer** | **Catalog Number** |
| --- | --- | --- | --- |
| phalloidin-488 | 1/100 | Molecular Probes | A12379 |
| phalloidin-TRICT | 1/100 | Sigma Aldrich | P1951 |

**Table S2. DNA sequence inserts of p73BS. p73 binding sites in yellow, bold underlined and green respectively; deleted bases in red (RS=restriction sites)**

| **Construct name** | **DNA sequence of inserts** | **Vector** | **5´RS** | **3´RS** |
| --- | --- | --- | --- | --- |
| MLCK WT p73 binding site | 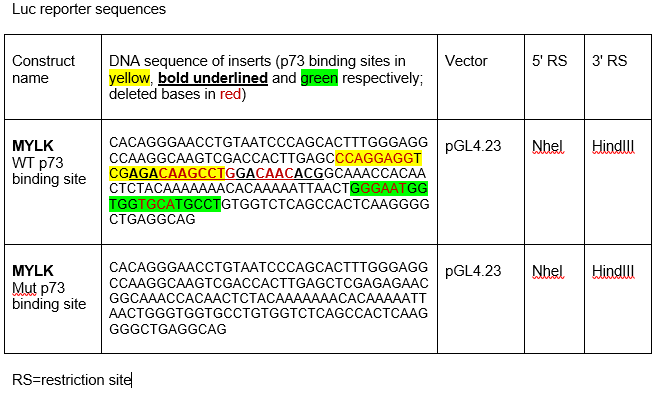 | pGL4.23 | NheI | HindIII |
| MLCK Mutated p73 binding site | 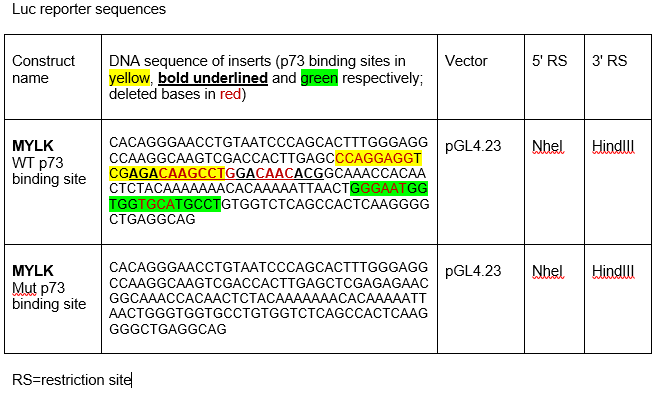 | pGL4.23 | NheI | HindIII |
