# Supplementary figures and images for: p73 regulates ependymal planar cell polarity by modulating actin and microtubule cytoskeleton
Source: Cell Death Dis. 2018 Dec 5;9(12):1183. doi: 10.1038/s41419-018-1205-6 (PMC6281643; doi:10.1038/s41419-018-1205-6)

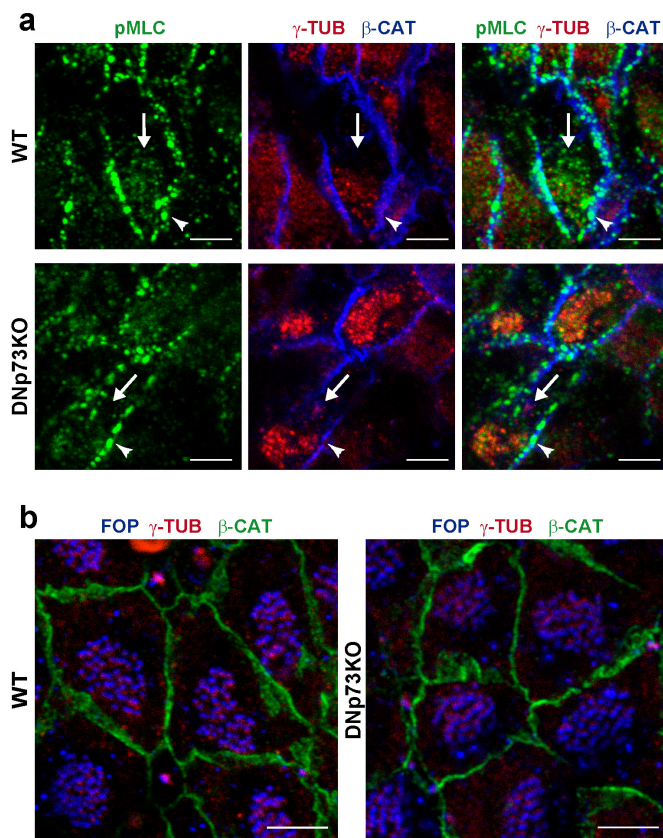

Supplementary 1. Fuertes-Alvarez *et al.*

Supplement: Supplementary file 3 — Supplementary Figure 1. Lack of DNp73 has no effect on PCP establishment or pMLC localization or rotational PCP Basal Bodies organization [file 41419_2018_1205_MOESM3_ESM.pdf]

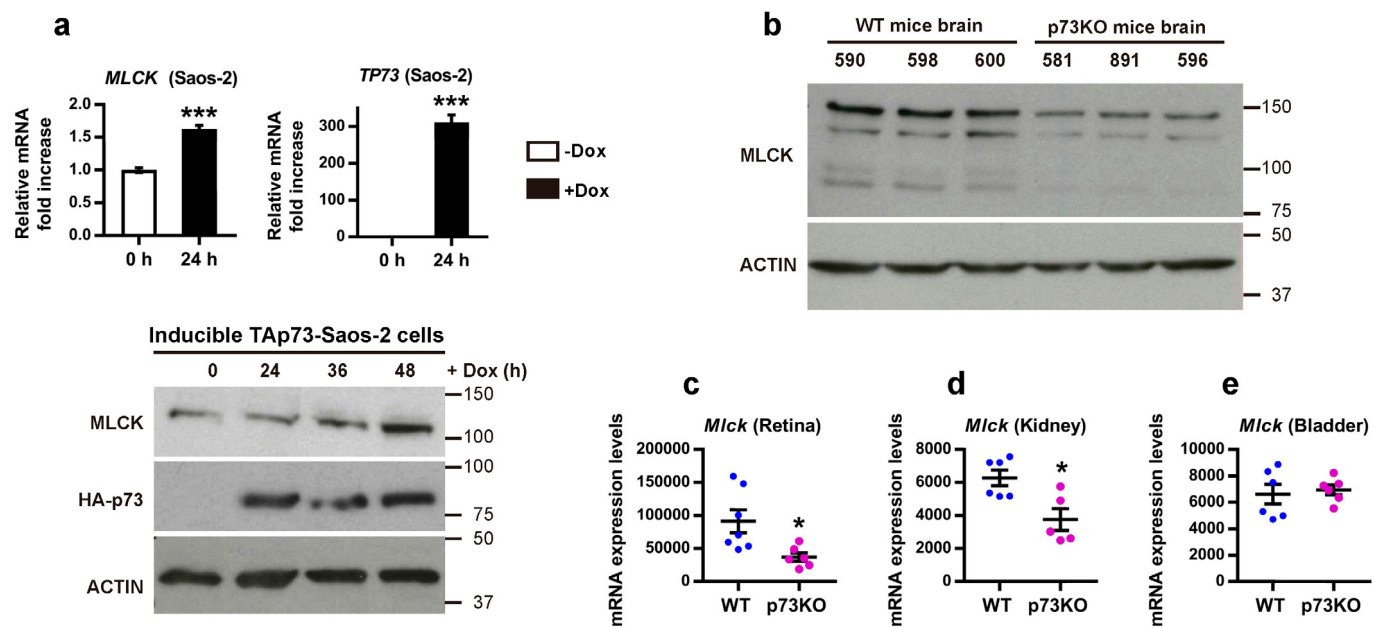

Supplementary 2. Fuertes-Alvarez *et al.*

Supplement: Supplementary file 4 — Supplementary Figure 2. p73 expression correlates with MLCK levels [file 41419_2018_1205_MOESM4_ESM.pdf]

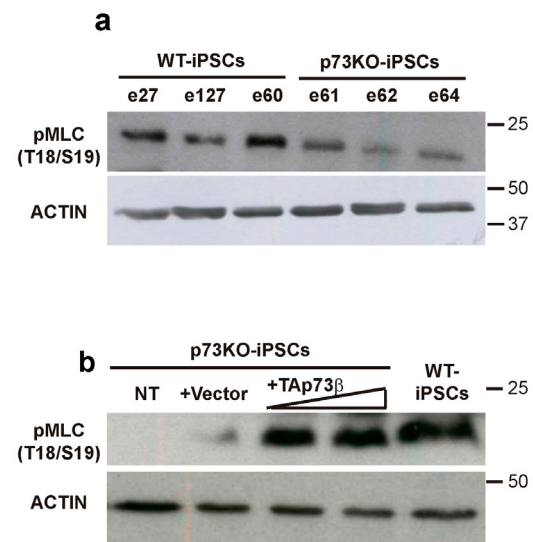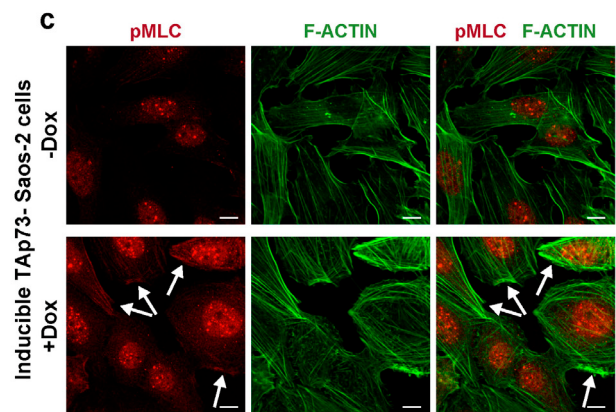

Supplementary 3. Fuertes-Alvarez *et al.*

Supplement: Supplementary file 5 — Supplementary Figure 3. TAp73 overexpression induces NMII activation [file 41419_2018_1205_MOESM5_ESM.pdf]
