# Supplementary material for: p73 regulates ependymal planar cell polarity by modulating actin and microtubule cytoskeleton
Source: Cell Death Dis. 2018 Dec 5;9(12):1183. doi: 10.1038/s41419-018-1205-6 (PMC6281643; doi:10.1038/s41419-018-1205-6)
Supplement: Supplementary file 6 — Supplementary Figure 4. TAp73 overexpression results in changes in “Golgi and MT organization” signaling pathways as well as Actin dynamics [file 41419_2018_1205_MOESM6_ESM.pdf]

a

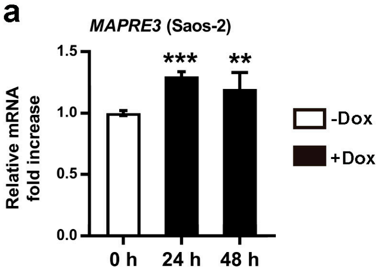

b

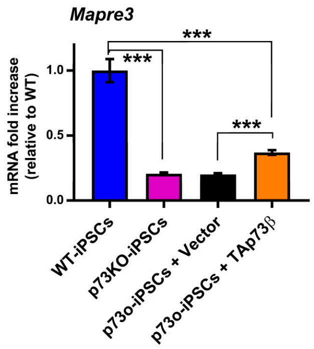

c

DAVID FUNCTIONAL ANNOTATION_CELLULAR COMPONENT				
**TERM**	**COUNTS**	**FOLD ENRICHMENT**	**FDR**	**HIGHLIGHTED GENES**
Golgi apparatus (GO:0005794)	202	1.59	6.19E-09	*AKAP 9, PDE4DIP, GOLGA1, GOLB1, GOLIM4*
Golgi membrane(GO:0000139)	143	1.65	1.11E-06	*GOLB1, GOLIM4*
Perinuclear region of cytoplasm (GO:0048471)	146	1.60	5.77E-06	*GOLGA1*
trans-Golgi network (GO:0005802)	43	2.15	0.00177361	*GOLGA1*
Actin cytoskeleton (GO:0015629)	60	1.87	0.00209022	*MLCK*
Lamellipodium (GO:0030027)	47	2.00	0.00514141	*MLCK*
Stress fiber (GO:0001725)	22	2.77	0.017675	*MLCK*

d

PANTHER FUNCTIONAL ANNOTATION_BIOLOGICAL PROCESS				
**TERMS**	**COUNTS**	**FOLD ENRICHMENT**	**FDR**	**HIGHLIGHTED GENES**
Regulation of protein localization to membrane (GO:1905475)	61	2.38	2,07E-06	*KIF5B, RAB11A*
Regulation of microtubule-based process (GO:0032886)	55	1.83	4,16E-03	*AKAP9*
Positive regulation of protein localization to membrane (GO:1905477)	45	2.65	1,31E-05	*KIF5B, RAB11A*
Regulation of protein localization to cell periphery (GO:1904375)	39	2.44	3,28E-04	*RAB11A, KIF5B*
Post-golgi vesicle-mediated transport (GO:0006892)	28	2.10	2,30E-02	*GOLPH3*
Regulation of microtubule polymerization (GO:0031113)	19	2.86	9,96E-03	*PDE4DIP, AKAP9*
Positive regulation of microtubule polymerization or depolymerization (GO:0031112)	13	2.96	3,28E-02	*PDE4DIP, AKAP9*
Positive regulation of microtubule polymerization (GO:0031116)	13	3.40	1,51E-02	*PDE4DIP, AKAP9*
Protein localization to Golgi apparatus (GO:0034067)	12	3.03	4,01E-02	*GOLPH3, RAB6A*
Regulation of actin filament-based process (GO:0032970)	106	2.00	1,17E-04	*MYLK3, MYH9, VANGL2, AKAP9*
Regulation of actin cytoskeleton organization (GO:0032956)	90	1.92	9,17E-03	*MYLK3, VANGL2*
Regulation of actin filament organization (GO:0110053)	75	2.02	1,88E-05	*ACTR2, LIMA1, PXN*
Regulation of actin filament bundle assembly (GO:0032231)	34	2.58	3,39E-04	*MYLK3, MYH9, VANGL2, AKAP9*
Regulation of actomyosin structure organization (GO:0110020)	33	2.68	3,05E-04	*MYLK3*
Regulation of stress fiber assembly (GO:0051492)	31	2.77	2,68E-04	*AMOT PXN*
Lamellipodium organization (GO:0097581)	17	2.73	2,21E-02	*GOLPH3*
Regulation of lamellipodium assembly (GO:0010591)	13	2.96	3,28E-02	*CAPZB*
Postsynaptic actin cytoskeleton organization (GO:0098974)	8	7.07	6,65E-03	*ACTB*

Supplementary 4. Fuertes-Alvarez *et al*.
